# Bayesian variable selection in sample selection models using spike-and-slab priors

**DOI:** 10.1007/s00180-026-01745-3

**Published:** 2026-05-13

**Authors:** Adam J. Iqbal, Emmanuel O. Ogundimu, F. Javier Rubio

**Affiliations:** 1 Department of Mathematical Sciences, University of Durham, Stockton Road, Durham, DH1 3LE UK; 2 Department of Statistical Science, University College London, 1-19 Torrington Place, London, WC1E 7HB UK

**Keywords:** Gibbs sampling, Heckman correction, Missing data, Prior elicitation, Scale mixtures

## Abstract

Sample selection models are a widely used approach for correcting bias caused by data that are missing not at random. Their formulation requires specifying the variables that influence the outcome and those that drive the selection process. This specification is often based on expert knowledge, which can result in the inclusion of irrelevant variables or the omission of important ones. Moreover, to avoid inferential problems such as practical non-identifiability, practitioners frequently impose *exclusion restrictions*, that is, model specifications in which certain variables predict selection but have no effect on the outcome of interest. A recent proposal employs adaptive LASSO to select the variables that enter into the outcome and selection equations, but its performance depends on the so-called covariance assumption, which can be violated in small to moderate samples. To address these challenges, we propose two families of spike-and-slab priors to conduct Bayesian variable selection in sample selection models. These prior structures allow for constructing a Gibbs sampler with tractable conditionals, which is scalable to the dimensions of practical interest. We illustrate the performance of the proposed methodology through a simulation study and present a comparison against adaptive LASSO and stepwise selection. We also provide two applications using publicly available real data.

## Introduction

Sample selection occurs when a portion of the sample is missing non-randomly, leading to a sample that is not representative of the population of interest. This is prevalent in medical and social sciences, where the outcome of interest is non-randomly selected (Heckman 1976, 1979; Wooldridge 2010). As a result, the sample becomes biased, potentially distorting the findings and generalizability of the results. Heckman (1976, 1979) introduced a regression model to correct for sample selection bias in the case where the outcome variable is continuous. The main idea is to formulate a two-equation regression model, one for the outcome process and one for the selection process, allowing for correlated errors under the assumption of bivariate normality. These kind of models are known as *sample selection* models (or Heckman correction). Heckman (1979) proposed a two-stage estimator of the parameters of sample selection models and established the consistency and asymptotic normality of such an estimator. After this influential development, several extensions have been proposed to alleviate challenges posed by the assumption of normality of the errors. These include van Hasselt (2011), who extended the outcome model to mixture-of-Gaussian error distributions. Marchenko and Genton (2012), who extended Heckman’s model by modelling the errors in the two-equation model using a bivariate-*t* distribution, which accounts for heavy tails, coupled with maximum likelihood estimation of the parameters. Ogundimu and Hutton (2016) assumed bivariate skew-normal errors, which account for departures from symmetry. Other extensions include the analysis of binary outcomes subject to sample selection bias. One of the challenges in these models is that the user must specify the variables that enter the outcome and selection models. Entering the same variables into both the outcome and selection submodels often leads to collinearity problems. It has also been shown that even with “exclusion restriction” rules - a covariate included in the selection but not the outcome - collinearity issues may appear in two-step estimators (Leung and Yu 2000). This challenge points to the need for formal tools for specifying the variables that enter the outcome and selection models.

Recently, the use of variable selection methods in the context of sample selection models has attracted considerable attention. For instance, Ogundimu (2022) used an Adaptive LASSO penalty to select the variables that enter the outcome and selection models. The distributional regression sample selection modelling introduced by Wiemann et al. (2022) allows for the use of Bayesian variable selection priors (shrinkage and spike-and-slab priors). However, Wiemann et al. (2022) only provide an implementation of their proposed model using a Metropolis within Gibbs sampler, which is computationally more expensive than a closed-form Gibbs sampler due to the need for iterative accept-reject Metropolis steps, and requires careful calibration of the priors for the additive components to ensure convergence. In a similar vein, Vera (2023) considered the use of shrinkage and spike-and-slab priors, but they restricted variable selection to only the selection equation, and their implementation similarly relies on a Metropolis within Gibbs sampler.

We propose two families of continuous spike-and-slab priors (George and McCulloch 1993; Tadesse and Vannucci 2021) to conduct Bayesian variable selection in sample selection models. The first class of priors allows for the use of scale mixtures of normals for the spike and slab components, which include Laplace and Student-*t* distributions. The second one is defined conditionally on the marginal variance of the outcome equation, allowing for easier calibration, and also includes scale mixtures of normals for the spike and slab components. We provide a detailed discussion on the calibration of both priors, taking into account the different nature of the outcome and selection models. We derive a closed-form Gibbs sampler for the posterior distribution of the parameters, under both classes of priors, which scales well to the dimensions of interest in practical applications. Posterior samples can be used to jointly select variables for both the selection and outcome equations, without the need for an exclusion restriction.

The remainder of this paper is organized as follows. In Sect. 2, we present a brief review of sample selection models and previous literature on Bayesian inference for these models. In Sect. 3, we present the proposed families of spike-and-slab priors and discuss the calibration of these priors. Section 4 presents details about the Gibbs sampler for the proposed prior structures. Section 5 presents a simulation study aimed at illustrating the performance of the proposed spike-and-slab priors for variable selection. We compare the performance of the proposed methodology with that of adaptive LASSO and forward selection. Indeed, a byproduct of this work is the implementation of stepwise variable selection methods for sample selection models. Section 6 presents real data applications using two popular data sets in the context of sample selection models and a sensitivity analysis for the hyperparameters. We conclude with a discussion and possible extensions in Sect. 7. An implementation and code to reproduce the results in this paper can be found at https://github.com/adam-iqbal/selection-spike-slab

## Sample selection models

Sample selection models address non-random missing outcomes by coupling a latent outcome equation with a latent selection index. Let $s_i\in \{0,1\}$ denote whether $y_i^{*}$ is observed ($s_i=1$) or missing ($s_i=0$), and let $s_i^{*}$ be the unobserved propensity for selection (e.g., an individual’s propensity to participate in the labor force). The model comprises

1 $$\begin{aligned} y^{*}_{i}= & \beta _{0} + \boldsymbol{x}_{i}^{\top }\boldsymbol{\beta } + \epsilon _{1,i} , \quad \text {(outcome)} \nonumber \\ s^{*}_{i}= & \alpha _{0} + \boldsymbol{w}_{i}^{\top }\boldsymbol{\alpha } + \epsilon _{2,i}, \quad \text {(selection)} \end{aligned}$$

with observed data $(y_i,s_i)$ related by $s_{i}={\mathbb {I}}(s^{*}_{i}>0)$ and

2 $$\begin{aligned} y_{i}= {\left\{ \begin{array}{ll} y_{i}^{*}, & s_{i}=1,\\ \text {not observed}, & s_{i}=0. \end{array}\right. } \end{aligned}$$

Here $\boldsymbol{x}_{i}=(x_{i,1},\ldots ,x_{i,p})^{\top }\in {\mathbb {R}}^{p}$ and $\boldsymbol{w}_{i}=(w_{i,1},\ldots ,w_{i,q})^{\top }\in {\mathbb {R}}^{q}$, $i=1,\ldots ,n$, are covariates; $\boldsymbol{\beta }=(\beta _{1},\ldots ,\beta _{p})^{\top }\in {\mathbb {R}}^{p}$ and $\boldsymbol{\alpha }=(\alpha _{1},\ldots ,\alpha _{q})^{\top }\in {\mathbb {R}}^{q}$ are regression coefficients; and $\beta _{0},\alpha _{0}$ are intercepts. In the classical sample selection model, the bivariate errors are assumed to be independent and identically distributed, following a bivariate normal distribution:

3 $$\begin{aligned} \begin{pmatrix} \varepsilon _{1,i} \\ \varepsilon _{2,i} \end{pmatrix} \sim {\mathcal {N}}\!\left( \begin{pmatrix} 0\\ 0 \end{pmatrix}, \begin{pmatrix} \sigma ^{2} & \sigma \rho \\ \sigma \rho & 1 \end{pmatrix} \right) , \end{aligned}$$

with $\sigma >0$ and $\rho \in (-1,1)$. In (3) the selection variance is fixed to 1 because only the sign of $s_i^{*}$ is observed, so the index scale is not identified (e.g., Greene 2018, Ch. 17; Wooldridge 2010, Ch. 15). The selection regression $s_i^{*}=\alpha _{0}+\boldsymbol{w}_{i}^{\top }\boldsymbol{\alpha }+\varepsilon _{2,i}$ implies $\Pr (s_i=1\mid \boldsymbol{w}_i)=\Phi (\alpha _{0}+\boldsymbol{w}_{i}^{\top }\boldsymbol{\alpha })$ (a probit model for non-missingness).

If $\rho =0$, selection is non-informative and the data is missing at random (MAR) given the covariates, and valid inference for the conditional distribution of $y^{*}$ given $\boldsymbol{x}$ can be based on the complete cases (or by adjusting for covariates). In this case the outcome error has zero mean in the selected sample, and ${\mathbb {E}}(\varepsilon _{1,i}\mid s_i=1)=0$. Thus, a standard regression on the observed data is consistent. If, in addition, the non-intercept terms in the selection equation coefficients (i.e., $\boldsymbol{\alpha }$) are zero, then selection depends only on an intercept, and in this case the data is missing completely at random (MCAR), and no adjustment is needed.

If $\rho >0$, we have positive selection bias and the implication is that unobserved factors that make selection more likely are positively correlated with unobserved factors that increase the outcome. So the selected sample tends to have a higher mean outcome than a random draw from the population. Conversely, there is negative selection when $\rho <0$. In general, when $\rho \ne 0$, the outcome error has a nonzero conditional mean among selected units, which is the source of the bias. Using the properties of the bivariate normal distribution, this conditional mean can be derived as:

$$\begin{aligned} {\mathbb {E}}(\varepsilon _{1,i}\mid s_i=1,\boldsymbol{x}_i,\boldsymbol{w}_i) = {\mathbb {E}}\!\left( \varepsilon _{1,i}\mid \varepsilon _{2,i} > -(\alpha _0+\boldsymbol{w}_i^{\top }\boldsymbol{\alpha })\right) = \sigma \,\rho \,\lambda \!\left( \alpha _0+\boldsymbol{w}_i^{\top }\boldsymbol{\alpha }\right) , \end{aligned}$$

where $\lambda (u)=\phi (u)/\Phi (u)$ is the inverse Mills ratio (IMR) and $\phi ,\Phi$ denote the standard normal probability density function (pdf) and cumulative distribution function (cdf). The IMR equals the mean of a standard normal truncated to $\{Z>-u\}$; it is strictly positive and strictly decreasing in *u* (indeed, $\lambda '(u)=-\lambda (u)\{u+\lambda (u)\}<0$). Larger selection propensity $u=\alpha _0+\boldsymbol{w}_i^\top \boldsymbol{\alpha }$ therefore implies a smaller correction term $\sigma \rho \,\lambda (u)$. Consequently,

4 $$\begin{aligned} {\mathbb {E}}(y_i^{*}\mid s_i=1,\boldsymbol{x}_i,\boldsymbol{w}_i) =\beta _0+\boldsymbol{x}_i^{\top }\boldsymbol{\beta }+\sigma \,\rho \,\lambda \!\left( \alpha _0+\boldsymbol{w}_i^{\top }\boldsymbol{\alpha }\right) . \end{aligned}$$

Beyond the mean, $\rho$ also affects dispersion in the selected sample. For the truncated bivariate normal one obtains $\textrm{Var}(\varepsilon _{1,i}\mid s_i=1,\boldsymbol{x}_i,\boldsymbol{w}_i) =\sigma ^2\!\left[ 1-\rho ^2\,\kappa (u_i)\right]$, $u_i:=\alpha _0+\boldsymbol{w}_i^\top \boldsymbol{\alpha }$, and $\kappa (u):=\lambda (u)\{\lambda (u)+u\}$. This implies that the selection process makes the observed data heteroscedastic, and the observed variance is always less than the true population variance $\sigma ^2$. The amount of this variance reduction depends on both the strength of the selection effect ($\rho$) and each observation’s propensity to be selected ($u_i$).

Equation (4) is the conditional expectation of the observed data and is the basis of the Heckman two-step method (Heckman 1979). In practice, a probit for $s_i$ on $\boldsymbol{w}_i$ provides estimates $({\hat{\alpha }}_0,\hat{\boldsymbol{\alpha }})$, from which $\widehat{\lambda }_i=\lambda ({\hat{\alpha }}_0+\boldsymbol{w}_i^{\top }\hat{\boldsymbol{\alpha }})$ is computed. One then estimates, on the selected sample $\{i:s_i=1\}$,

$$\begin{aligned} y_i \;=\; \beta _0+\boldsymbol{x}_i^{\top }\boldsymbol{\beta }\;+\; \delta \,\widehat{\lambda }_i \;+\; \text {error}, \end{aligned}$$

where the coefficient ${\hat{\delta }}$ estimates $\sigma \rho$.

The log-likelihood of the parameters for the sample selection model (1)–(3) is given by (Toomet and Henningsen 2008)

$$\begin{aligned} \ell (\alpha _{0}, \beta _{0}, \boldsymbol{\alpha }, \boldsymbol{\beta }, \sigma , \rho )= & \sum _{\{s_{i}=0\}}\log \Phi (-\alpha _{0} - \boldsymbol{w}_{i}^{\top }\boldsymbol{\alpha })\\+ & \sum _{\{s_i=1\}} \Bigg ( \log \Phi \left( \frac{\alpha _{0} + \boldsymbol{w}_{i}^{\top }\boldsymbol{\alpha } + \rho (y_{i} - \beta _{0} - \boldsymbol{x}_{i}^{\top }\boldsymbol{\beta })/\sigma }{\sqrt{1-\rho ^{2}}}\right) \\ & \qquad \quad - \frac{1}{2}\left( \frac{y_{i} - \beta _{0} - \boldsymbol{x}_{i}^{\top }\boldsymbol{\beta }}{\sigma }\right) ^{2} - \log (\sigma ) - \frac{1}{2}\log (2\pi ) \Bigg ) . \end{aligned}$$

Maximum likelihood estimators of the parameters can be found by maximizing the log-likelihood function using any general-purpose optimization method.

A central challenge is specifying covariates for the outcome ($\boldsymbol{x}_i$) and selection ($\boldsymbol{w}_i$) equations. In practice, those variables that affect the outcome also affect the selection, so $\boldsymbol{x}_i$ is often chosen either as a subset of, or equal to, $\boldsymbol{w}_i$. While the model is identified in principle even if $\boldsymbol{x}_i$ and $\boldsymbol{w}_i$ coincide, practical estimation is often hindered by flat likelihood regions and potential misspecification. Empirical identification is therefore substantially stronger when an *exclusion restriction* (ER) is available (e.g., Chib and G.E. and I. Jeliazkov. 2009; Wiesenfarth and Kneib 2010; Vella 1998). In many applications - with samples typically in the hundreds to low thousands (e.g., Mroz, $n{=}753$, Ambulatory expenditures data, $n{=}3,328$) - the same predictors plausibly influence both selection and outcome. This overlap, coupled with the fact that the inverse Mills ratio $\lambda (u)$ is nearly linear over wide ranges of its support, can induce severe multicollinearity, particularly in two-step implementations (Puhani 2000; Leung and Yu 2000).

When theory points to identical covariates, a common reaction is a “mad” search for an ER, but adding extraneous regressors solely for identification risks specification error (Sartori 2003). We therefore propose a joint, data-adaptive variable-selection approach across both equations. This framework allows plausible exclusions to be formally tested rather than imposed, while shrinkage helps mitigate collinearity when $\boldsymbol{x}_i$ and $\boldsymbol{w}_i$ necessarily overlap.

In the Bayesian framework, van Hasselt (2011) showed that it is possible to construct a tractable Gibbs sampler for the model defined by equations (1)-(2) by using the reparametrization ${\tilde{\sigma }}^{2} = \sigma ^{2}(1 - \rho ^{2})$ and ${\tilde{\rho }} = \rho \sigma$. The conditional distributions for $(s^{*}_{i}, y^{*}_{i})$ under this reparametrization are

5 $$\begin{aligned} \begin{aligned} s^{*}_{i} \mid \boldsymbol{\theta }&\sim N(\alpha _{0} + \boldsymbol{w}^{\top }_{i}\boldsymbol{\alpha }, 1) \,,\\ s^{*}_{i} \mid \{y^{*}_{i}, \boldsymbol{\theta }\}&\sim N(\alpha _{0} + \boldsymbol{w}^{\top }_{i}\boldsymbol{\alpha } + \frac{{\tilde{\rho }}}{{\tilde{\sigma }}^{2} + {\tilde{\rho }}^{2}}(y_{i} - \beta _{0} - \boldsymbol{x}^{\top }_{i}\boldsymbol{\beta }), \frac{{\tilde{\sigma }}^{2}}{{\tilde{\sigma }}^{2} + {\tilde{\rho }}^{2}})\,,\\ y^{*}_{i} \mid \{s^{*}_{i}, \boldsymbol{\theta }\}&\sim N\left( \beta _{0} + \boldsymbol{x}^{\top }_{i}\boldsymbol{\beta } + {\tilde{\rho }}(s^{*}_{i} - \alpha _{0} - \boldsymbol{w}_{i}^{\top }\boldsymbol{\alpha }\right) , {\tilde{\sigma }}^{2})\,. \end{aligned} \end{aligned}$$

where $\boldsymbol{\theta } = (\beta _{0},\boldsymbol{\beta }^{\top },\alpha _{0},\boldsymbol{\alpha }^{\top },{\tilde{\rho }},{\tilde{\sigma }}^{2})^{\top }$. Full details about the Gibbs sampler, and the corresponding priors, are presented by van Hasselt (2011) and reproduced in Appendix C for completeness. The major advantage of this Gibbs sampler is that every conditional distribution is in closed-form, allowing for fast simulations. The sampler does rely on $(p+1) \times (p+1)$ and $(q+1) \times (q+1)$ matrix inversions, but it is rare for the problems of interest in sample selection literature to exhibit dimensions high enough for this to pose a serious concern. The Gibbs sampler also requires sampling from *n* truncated normal random variables at each step, but this can be done relatively fast using available methods and R packages (Geweke 1991). We will capitalize on van Hasselt (2011) to develop a Gibbs sampler in Sect. 4 for the priors proposed in Sect. 3.

## Spike-and-slab prior formulation

Our aim is to perform Bayesian variable selection in sample selection models of type (1). To this end, let us define the variable inclusion indicators as follows. For $j = 1, \ldots , p$, let $\gamma ^{O}_{j} = 1$ if $\beta _{j}$ is included in the outcome model, and $\gamma ^{O}_{j} =0$ otherwise. Similarly, for $k = 1, \ldots , q$, let $\gamma ^{S}_{k} = 1$ if $\alpha _{k}$ is included in the selection model, and $\gamma ^{S}_{k} = 0$ otherwise. Next, we present the two proposed classes of priors. The first class of priors contains continuous spike-and-slab priors with scale mixture of normal components (George and McCulloch 1993). The second class of priors is defined conditionally on the marginal variance of the outcome model. Finally, we present a discussion on elicitation for each class of priors.

### Priors

The Class I spike-and-slab prior (George and McCulloch 1993) is defined by the structure:

6 $$\begin{aligned} \begin{aligned} \boldsymbol{\beta } \mid \{\boldsymbol{\gamma ^{O}}, \boldsymbol{v^{O}}\}&\sim \prod _{j=1}^{p}\left( (1-\gamma ^{O}_{j})N(0,\tau _{0,\beta }^{2}v^{O}_{j}) + \gamma _{j}^{O}N(0,\tau _{1,\beta }^{2}v^{O}_{j})\right) \,\\ \boldsymbol{\alpha } \mid \{\boldsymbol{\gamma ^{S}},\boldsymbol{v^{S}}\}&\sim \prod _{k=1}^{q}\left( (1-\gamma ^{S}_{k})N(0,\tau _{0,\alpha }^{2}v^{S}_{k}) + \gamma _{k}^{S}N(0,\tau _{1,\alpha }^{2}v^{S}_{k})\right) \,, \end{aligned} \end{aligned}$$

where $v_{j}^{O}$ and $v_{k}^{S}$ are positive random variables, allowing for scale mixtures of normal distributions as priors, such as Laplace and Student-*t* distributions which have both seen use for spike-and-slab priors in other contexts (Ročková and George 2018; Scheipl et al. 2012). Formally, we let

7 $$\begin{aligned} p(v_{j}^{O} \mid \gamma _{j}^{O}) = (1-\gamma _{j}^{O})\pi _{0,\beta }(v_{j}^{O}) + \gamma _{j}^{O}\pi _{1,\beta }(v_{j}^{O}) \,, \end{aligned}$$

where $\pi _{0,\beta }$ and $\pi _{1,\beta }$ are probability density functions with positive support. The distribution of $v_{k}^{S} \mid \gamma _{k}^{S}$ is defined analogously. Note that conditional on $\gamma _{j}^{O}$, only one of the components in (7) is active, so marginalizing (6) over $\boldsymbol{v^{O}}$ and $\boldsymbol{v^{S}}$ results in the components being different scale mixtures of normals, depending on the choices of $\pi _{0,\beta }, \pi _{1,\beta }, \pi _{0,\alpha }$ and $\pi _{1,\alpha }$. Hence, this formulation allows for different distributions for the spike and slab components. For instance, one may desire to use a heavier-tailed distribution for the slab component than the spike component, such as using a Laplace slab with a normal spike, or alternatively using a prior with more mass concentrated around zero for the spike. As shown in Sect. 4, the only additional complexity this adds to the sampling procedure is an extra step sampling $v_{j}^{O} \mid (\gamma _{j}^{O}, \beta _{j})$ and $v_{k}^{S} \mid (\gamma _{k}^{S}, \alpha _{k})$. If these conditional distributions are closed form (as they are for Laplace and t-distribution priors) or easy to sample from, scale mixture priors can be seamlessly integrated into the sampling algorithm with minimal adverse effect on computational time.

The Class II spike-and-slab prior is defined by the structure:

8 $$\begin{aligned} \begin{aligned} \boldsymbol{\beta } \mid \{\boldsymbol{\gamma ^{O}}, \boldsymbol{v^{O}}, {\tilde{\sigma }}^{2}\}&\sim \prod _{j=1}^{p}\left( (1 - \gamma ^{O}_{j})N(0,\tau _{0,\beta }^{2}v^{O}_{j}{\tilde{\sigma }}^{2}) + \gamma _{j}^{O} N(0,\tau _{1,\beta }^{2}v^{O}_{j}{\tilde{\sigma }}^{2})\right) \,,\\ \boldsymbol{\alpha } \mid \{\boldsymbol{\gamma ^{S}},\boldsymbol{v^{S}}\}&\sim \prod _{k=1}^{q}\left( (1-\gamma ^{S}_{k})N(0,\tau _{0,\alpha }^{2}v^{S}_{k}) + \gamma _{k}^{S}N(0,\tau _{1,\alpha }^{2}v^{S}_{k})\right) \,, \end{aligned} \end{aligned}$$

with $\boldsymbol{v^{O}}$ and $\boldsymbol{v^{S}}$ defined as before. In sample selection models, the variance of the observed outcomes is ${\tilde{\sigma }}^{2}$, as shown in equation (5). Choosing the marginal variance $\sigma ^{2} = {\tilde{\sigma }}^{2} + {\tilde{\rho }}^{2}$ would not lead to a natural expression for the posterior of ${\tilde{\sigma }}^{2}$, whereas ${\tilde{\sigma }}^{2} = \sigma ^{2}(1 - \rho ^{2})$ does.

The advantage of Class II priors over Class I is that the magnitude of variables is only considered relative to the unexplained variance. This is a common strategy in the standard linear regression context (Louzada et al. 2023). The cost of using ${\tilde{\sigma }}^{2}$ instead of $\sigma ^{2}$ is that the observed variance is less than the true variance, with ${\tilde{\sigma }}^{2}$ being shrunk dependent on $\rho$. When $\rho = 0$, ${\tilde{\sigma }}^{2}$ collapses to $\sigma ^{2}$ as expected. For larger magnitudes of $\rho$, the difference is greater. For instance, when $\rho = 0.7$, ${\tilde{\sigma }}^{2} = 0.49\sigma ^{2}$, shrinking the slab variance by a factor of about a half, which could lead to higher false positive rates.

The priors for the remaining parameters, common to both classes of priors, are specified as follows. We let $\gamma ^{O}_{j}$ and $\gamma ^{S}_{k}$ follow *i.i.d.* Bernoulli distributions with parameter *r*. We let $r \sim Beta(a_{0},b_{0})$, so that the priors on $\boldsymbol{\gamma }^{O}$ and $\boldsymbol{\gamma }^{S}$ are Beta-Binomial. One could also consider different *r*’s for the outcome and selection models, aiming at separating the penalties on the corresponding model sizes. We do not pursue this option in this work, but this could be explored as a future extension of this work. We further assume ${\tilde{\rho }} \mid {\tilde{\sigma }} \sim N(0, \tau {\tilde{\sigma }}^{2})$, and ${\tilde{\sigma }}^{2} \sim \text {IG}(c,d)$, where $\text {IG}$ denotes an inverse gamma distribution. For the intercepts, we use weakly informative priors $\beta _{0} \sim N(0,\eta ^{O}v^{O}_{0})$ and $\alpha _{0} \sim N(0,\eta ^{S}v^{S}_{0})$, with large values of $\eta ^{S}$ and $\eta ^{O}$, and $v^{O}_{0} \sim \pi _{I,\beta }$ and $v^{S}_{0} \sim \pi _{I,\alpha }$ to allow for scale mixtures of normals as priors for the intercepts.

### Prior calibration

Posterior inference is sensitive to the choice of both the Beta-Binomial parameters and the spike and slab variances (Tadesse and Vannucci 2021). In this section we discuss intuitive choices that we found to work reasonably well in practice. The performance of this strategy will be illustrated through a simulation study in Sect. 5 along with a sensitivity analysis in Sect. 6.3.

**Fig. 1:**
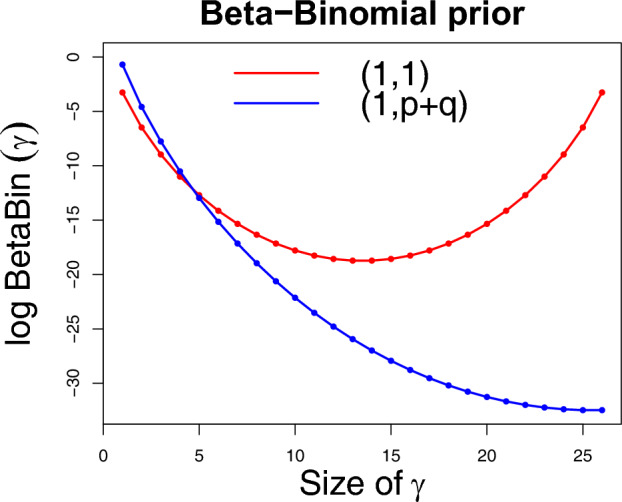
Beta-Binomial log-prior for full model size 25 under two different calibrations. The *x*-axis shows model size while the *y*-axis shows the prior probability of an individual model of that size

**Beta-Binomial hyperparameters:** For the Beta-Binomial part of the model, $r \sim Beta(a_{0}, b_{0})$, a natural choice is $a_{0} = b_{0} = 1$, which induces a uniform prior on model size. This choice is reasonable in sparse scenarios, but it allows for ${\mathbb {E}}(r \mid \boldsymbol{\gamma ^{O}},\boldsymbol{\gamma ^{S}}) > 0.5$, that is the prior inclusion probability to be greater than 0.5. In Fig. 1, which displays the Beta-Binomial prior over model sizes, the prior mass decreases as the model size increases, but begins to rise again for sizes greater than approximately $\frac{p+q}{2}$. This increase reflects the multiplicity of larger models, which receive greater individual prior probability due to the smaller number of possible configurations (analogous to models with small number of variables). Another interpretation can be found by looking at the conditional posterior of the Bernoulli parameter *r* in Sect. 4. Under a Beta prior with parameters (1, 1), if more than half of the variables are included, the expected value of *r* for the next draw exceeds 0.5, inducing a bias towards including irrelevant variables.

An alternative is using $a_{0} = 1, b_{0} = p+q$, so that ${\mathbb {E}}(r \mid \boldsymbol{\gamma ^{O}},\boldsymbol{\gamma ^{S}}) \le 0.5$ in every case except the full model. This can also be seen in Fig. 1: the prior includes only the left half of the parabola, so that it is monotonically decreasing. The trade-off is that significantly more sparsity is induced. As such, the choice of Beta-Binomial prior parameters depends on the context. In cases where significant sparsity is present, the choice $a_{0}=b_{0}=1$ works well and has a greater chance of including small effects. If close to (or more than) half the variables may be significant, the prior $a_{0}=1,b_{0}=p+q$ is a more sensible choice, at the cost of potentially shrinking small effects. Regardless of our recommendations, our implementation allows the user to choose their desired specification of Beta-Binomial hyperparameters.

**Spike-and-slab hyperparameters:** Continuous spike-and-slab priors are sensitive to the choice of variances, so we give particular importance to their calibration. We calibrate the variances for the normal prior case, as calibration for scale mixtures of normals will depend on the scale distribution and their tail-weight. While theoretical results are not available for sample selection models, we take inspiration from Narisetty and He (2014) and impose $\tau _{0,\alpha } \rightarrow 0$ as $n \rightarrow \infty$ (and similarly for $\tau _{0,\beta }$). In sparse situations, the Beta-Binomial prior imposes greater bias against small effects as *p* and *q* increase. This is because each $\gamma ^{O}_{j}$ and $\gamma ^{S}_{k}$ are drawn from a Bernoulli with the parameter *r* having a Beta hyperprior over it. The posterior for *r* depends on the number of variables included - the less variables included, the smaller the expected value of $r \mid \{\boldsymbol{\gamma ^{O}}, \boldsymbol{\gamma ^{S}}\}$. For very sparse situations, *r* is shrunk to small values, so that the prior inclusion probability becomes very small. As a result, the Beta-Binomial prior avoids the multiple testing problem when there are many irrelevant variables, but for small sample sizes it can lead to the exclusion of small effects more often than desired. To partially offset this, we propose the choice $\tau _{0,\alpha } = 1/\sqrt{nc_{p}}$ and $\tau _{0,\beta } = 1/\sqrt{nc_{q}}$, with $c_{p}$ and $c_{q}$ increasing with respect to *p* and *q*. This puts less density away from 0 as the dimension increases, and empirically we found that $c_{p} = p$ and $c_{q} = q$ were relatively effective as heuristic choices. The factor $1/\sqrt{n}$ is motivated by theoretical results for linear regression models for spike-and-slab priors (Narisetty and He 2014; Narisetty et al. 2019).

We similarly choose $\tau _{1,\alpha }$ and $\tau _{1,\beta }$ in line with previous work on spike-and-slab priors. For the probit equation, we choose $\tau _{1,\alpha } = C_{\alpha }$, a constant. This is in line with the choice made by Narisetty et al. (2019) for logistic regression. We choose $C_{\alpha } = \sqrt{3}/\pi$ to match the probit scale. For the linear part of the model, we use $\tau _{1,\beta } = C_{\beta }(\log (n))^{1/2}$, which was used by Narisetty and He (2014) in their simulations. The discussion in Section 3.4 of Tadesse and Vannucci (2021) suggests that for this choice, as $n \rightarrow \infty$, the probability of including a non-zero effect will converge to 1. This would be true for a fixed choice $\tau _{1,\beta }$ independent of *n* as well, but the elicitation of $\tau _{1,\beta }$ is dependent on sample size. Even for moderately large sample sizes, a small choice of $\tau _{1,\beta }$ can shrink large coefficients and negatively affect the inference. But for small sample sizes, large $\tau _{1,\beta }$ will exclude small effects, losing sensitivity. Since this is not as much of an issue for moderately large sample sizes, we suggest a slab variance proportional to $\log (n)$.

The elicitation of $C_{\beta }$ still depends on the context, and our default choice is derived as follows. Suppose that $\tau _{0,\beta } = 1/\sqrt{n}$ and assume that $r = 0.5$. For a small effect of magnitude 0.25, there is variability in the posterior sampling, so we wish to ensure that we include marginally smaller effects, say of magnitude 0.15, with high probability, but not effects significantly smaller. With $\tau _{1,\beta } = 0.5$, the probability of including a coefficient sample of magnitude 0.15 is approximately $96\%$. But an effect size of 0.05 only has a $14\%$ probability of inclusion. As a result, we recommend the heuristic choice of $C_{\beta } = \left( 4\log (500)\right) ^{-1/2}$.

**Table 1:** Summary of prior elicitations used in this paper for the spike-and-slab prior and Beta-Binomial prior

Parameter	$\tau _{0,\beta }$	$\tau _{0,\alpha }$	$\tau _{1,\beta }$	$\tau _{1,\alpha }$	Beta-Binomial
Elicitation	$(np)^{-0.5}$	$(nq)^{-0.5}$	$0.5(\log (n)/\log (500))^{-1/2}$	$\sqrt{3}/\pi$	Either (1, 1) or $(1,p+q)$

Table 1 summarizes the choices of prior parameters used in this paper. It should be noted that while these provide a reasonable trade-off between including small effects and excluding spurious ones, it may be desirable to focus on one over the other. For instance, if there is no interest in including small effects, one could use a larger value for $C_{\beta }$ such as 1 to improve specificity. The sensitivity analysis in Sect. 6.3 shows that the prior elicitation is not particularly sensitive to moderate perturbations in the spike-and-slab parameters, so prioritizing minimizing false positives should be achieved by setting the slab variance substantially larger.

**Other parameters:** We do not directly define priors on $\rho$ and $\sigma ^{2}$, but indirectly through ${\tilde{\rho }}$ and ${\tilde{\sigma }}^{2}$. The prior on ${\tilde{\sigma }}^{2}$ is $\text {IG}(c,d)$, with $c, d > 0$ positive constants, and the prior on ${\tilde{\rho }}$ is $N(0,\tau {\tilde{\sigma }}^{2})$ where $\tau > 0$ is a positive constant. We let the prior on ${\tilde{\rho }}$ depend on ${\tilde{\sigma }}^{2}$ so that the induced prior on $\rho$ has its density approach zero as $\rho \rightarrow \pm 1$. Choosing $c = d = 1$ leads to an induced prior on $\sigma ^{2}$ with similar behaviour to an Inverse Gamma. The choice of $\tau$ in the prior for ${\tilde{\rho }}$ has little impact on the posterior inference for $\boldsymbol{\gamma ^{S}},\boldsymbol{\gamma ^{O}},\alpha _{0},\beta _{0},\boldsymbol{\alpha },\boldsymbol{\beta }$ and $\sigma ^{2}$. However, it does have an impact on the posterior inference of $\rho$. The induced prior on $\rho$ depends on the choice of $\tau$: for small values such as $\tau = 0.5$, it behaves similarly to a uniform distribution, while for larger values like $\tau = 5$, it behaves similarly to a *Beta*(1/2, 1/2) distribution. Empirically we found that $\tau =5$ leads to more accurate posterior medians of $\rho$ than $\tau = 0.5$. See Appendix B for a further discussion.

## Gibbs samplers

In this section, we formulate Gibbs samplers for the sample selection model (1) coupled with the prior structures proposed in Sect. 3. After initializing all model parameters,

**Step 1.**: For $i = 1, \ldots , n$, sample from $\boldsymbol{s^{*}}$, where $s^{*}_{i} \mid \{y_{i}, s_{i}, \alpha _{0}, \beta _{0}, \boldsymbol{\alpha }, \boldsymbol{\beta }, {\tilde{\rho }}, {\tilde{\sigma }}\}$ is distributed according to
  $$\begin{aligned} {\left\{ \begin{array}{ll} \mathcal{T}\mathcal{N}_{(-\infty , 0)}(\alpha _{0} + \boldsymbol{w}_{i}^{\top }\boldsymbol{\alpha }, 1), & \text {if } y_{i} \text { is missing},\\ \mathcal{T}\mathcal{N}_{(0,\infty )}\left( \alpha _{0} + \boldsymbol{w}_{i}^{\top }\boldsymbol{\alpha } + \frac{{\tilde{\rho }}}{{\tilde{\rho }}^{2} + {\tilde{\sigma }}^{2}}(y_{i} - \beta _{0} - \boldsymbol{x}_{i}^{\top }\boldsymbol{\beta }), \frac{{\tilde{\sigma }}^{2}}{{\tilde{\rho }}^{2} + {\tilde{\sigma }}^{2}}\right) , & \text {otherwise}, \end{array}\right. } \end{aligned}$$
  where $\mathcal{T}\mathcal{N}_{(a,b)}$ denotes a truncated normal distribution on (*a*, *b*).
**Step 2.**: Sample $(\alpha _{0},\boldsymbol{\alpha }^{\top })^{\top }$ from $N(\boldsymbol{a^{*}}, \boldsymbol{A^{*}})$.
**Step 3.**: Jointly sample $(\beta _{0}, \boldsymbol{\beta }^{\top }, {\tilde{\rho }})^{\top }$ from $N(\boldsymbol{b^{*}}, \boldsymbol{B^{*}})$.
**Step 4.**: Sample ${\tilde{\sigma }}^{2}$ from $\text {IG}(c^{*},d^{*})$, where
  $$\begin{aligned} c^{*}&= c + \frac{1}{2}\left( 1 + \sum _{i=1}^{n} s_{i}\right) \, ,\\ d^{*}&= d + \frac{{\tilde{\rho }}^{2}}{2\tau } + \frac{1}{2}\sum _{\{i: s_{i}=1\}}\left( y_{i} - \beta _{0} -\boldsymbol{x}_{i}^{\top }\boldsymbol{\beta } - {\tilde{\rho }}(s^{*}_{i} - \alpha _{0} - \boldsymbol{w}_{i}^{\top }\boldsymbol{\alpha })\right) ^{2} \, . \end{aligned}$$
**Step 5.**: For $j = 1, \ldots , p$, sample $\gamma ^{O}_{j} \mid \{\beta _{j}, v^{O}_{j}\} \sim Ber(r^{O}_{j})$ where
  $$\begin{aligned} r^{O}_{j} = \frac{r\phi \left( \beta _{j}/\left( \tau _{1,\beta }\sqrt{v^{O}_{j}}\right) \right) \pi _{1,\beta }(v^{O}_{j})}{r\phi \left( \beta _{j}/\left( \tau _{1,\beta }\sqrt{v^{O}_{j}}\right) \right) \pi _{1,\beta }(v^{O}_{j}) + (1-r)\phi \left( \beta _{j}/\left( \tau _{0,\beta }\sqrt{v^{O}_{j}}\right) \right) \pi _{0,\beta }(v^{O}_{j})} \,. \end{aligned}$$
**Step 6.**: For $k = 1, \ldots , q$, sample $\gamma ^{S}_{k} \mid \{\alpha _{k}, v^{S}_{k}\} \sim Ber(r^{S}_{k})$ where
  $$\begin{aligned} r^{S}_{k} = \frac{r\phi \left( \alpha _{k}/\left( \tau _{1,\alpha }\sqrt{v^{S}_{k}}\right) \right) \pi _{1,\alpha }(v^{S}_{k})}{r\phi \left( \alpha _{k}/\left( \tau _{1,\alpha }\sqrt{v^{S}_{k}}\right) \right) \pi _{1,\alpha }(v^{S}_{k}) + (1-r)\phi \left( \alpha _{k}/\left( \tau _{0,\alpha }\sqrt{v^{S}_{k}}\right) \right) \pi _{0,\alpha }(v^{S}_{k})} \,. \end{aligned}$$
**Step 7.**: Sample $r \mid \{\boldsymbol{\gamma ^{O}}, \boldsymbol{\gamma ^{S}}\} \sim Beta(a_{1},b_{1})$, where
  $$\begin{aligned} a_{1}&= a_{0} + \sum _{j=1}^{p} \gamma ^{O}_{j} + \sum _{k=1}^{q} \gamma ^{S}_{k} \, ,\\ b_{1}&= b_{0} + p + q - \sum _{j=1}^{p} \gamma ^{O}_{j} - \sum _{k=1}^{q} \gamma ^{S}_{k} \, . \end{aligned}$$
**Step 8.**: For $j = 1, \ldots , p$, sample $v^{O}_{j} \mid \{\gamma ^{O}_{j}, \beta _{j}\}$ from
  $$\begin{aligned} p(v^{O}_{j} \mid \gamma ^{O}_{j}, \beta _{j}) \propto (1-\gamma ^{O}_{j})\phi \left( \frac{\beta _{j}}{\tau _{0,\beta }\sqrt{v^{O}_{j}}}\right) \pi _{0,\beta } (v^{O}_{j}) + \gamma ^{O}_{j}\phi \left( \frac{\beta _{j}}{\tau _{1,\beta }\sqrt{v^{O}_{j}}}\right) \pi _{1,\beta } (v^{O}_{j}) \,, \end{aligned}$$
  where $\pi _{0,\beta }$ and $\pi _{1,\beta }$ are the mixing distributions.
**Step 9.**: For $k = 1, \ldots , q$, sample $v^{S}_{k} \mid \{\gamma ^{S}_{k}, \alpha _{k}\}$ from
  $$\begin{aligned} p(v^{S}_{k} \mid \gamma ^{S}_{k}, \alpha _{k}) \propto (1-\gamma ^{S}_{k})\phi \left( \frac{\alpha _{k}}{\tau _{0,\alpha }\sqrt{v^{S}_{k}}}\right) \pi _{0,\alpha } (v^{S}_{k}) + \gamma ^{S}_{k}\phi \left( \frac{\alpha _{k}}{\tau _{1,\alpha }\sqrt{v^{S}_{k}}}\right) \pi _{1,\alpha } (v^{S}_{k}) \,; \end{aligned}$$
  where $\pi _{0,\alpha }$ and $\pi _{1,\alpha }$ are the mixing distributions.
**Step 10.**: Sample $v^{O}_{0} \sim \phi (\beta _{0}/(\sqrt{\eta ^{O}v^{O}_{0}}))\pi _{I,\beta }(v^{O}_{0})$ and $v^{S}_{0} \sim \phi (\alpha _{0}/(\sqrt{\eta ^{S}v^{S}_{0}}))\pi _{I,\alpha }(v^{S}_{0})$.

The exact expressions for $\boldsymbol{a^{*}}, \boldsymbol{A^{*}}, \boldsymbol{b^{*}}$ and $\boldsymbol{B^{*}}$ can be found in Appendix C. Steps 1 through 7 have distributions that are well-known and can easily be sampled from. Steps 8, 9 and 10 depend on the choice of scale distributions $\pi _{0,\alpha }, \pi _{1,\alpha }, \pi _{0,\beta }$ and $\pi _{1,\beta }$. There is no closed form expression for a general scale mixing distribution, but certain choices lead to closed form sampling for these steps. A Laplace prior can be used by choosing the mixing distribution to be $\text {Exp}(1/2)$: in this case, the posterior distribution to sample from takes the form of an Inverse Gaussian (after conditioning in Step 8/9). A Student-*t* prior can be used by choosing the mixing distribution to be Inverse Gamma, which is conjugate to the normal likelihood. In these cases and the normal case (by choosing the mixing distributions to be point masses at 1), the sampler is in closed form.

The output of the above procedure will be a sample from the posterior $(\boldsymbol{\alpha }^{\top }, \boldsymbol{\beta }^{\top }, {\boldsymbol{\gamma ^{S}}}^{\top }, {\boldsymbol{\gamma ^{O}}}^{\top }, {\tilde{\sigma }}^{2}, {\tilde{\rho }})^{\top } \mid \{\boldsymbol{y}, \boldsymbol{s}\}$. While the sampler still relies on $(p+1) \times (p+1)$ and $(q+1) \times (q+1)$ matrix inversions, the dimensions are usually not so big in applications of interest that this is problematic.

## Simulation study

In this section, we present a simulation study that aims at illustrating how the prior calibration performs as the dimension increases in highly sparse situations, for small and moderately large samples and typical levels of missingness in the data. We compare the performance of the Gibbs samplers to alternatives such as Adaptive LASSO and stepwise selection.

### Simulation scenarios

The true model is of the form in (1) with $\boldsymbol{\alpha } = \left( 0.5, 1, 1.5, 0, \ldots , 0\right) /\sqrt{2} \in {\mathbb {R}}^{q}$, $\boldsymbol{\beta } = \left( 0.25, 0.5, 1, 0, \ldots , 0\right) \in {\mathbb {R}}^{p}$, that is 3 active variables and $p - 3$ spurious variables, and $p = q$. The effect sizes are chosen to represent a small, medium and large effect respectively. The effect sizes for the probit equation are in line with Certo et al. (2016). The intercept $\alpha _{0}$ is chosen dependent on the simulated covariates, so that the expected proportion of missing data is 0.3. We choose $\beta _{0} = 0.5$ and $\sigma = 1$. We generated *n* covariates $\boldsymbol{w}_{i} \in {\mathbb {R}}^{q}$ such that their marginal distributions are standard normal and $\text {cov}(w_{j},w_{k}) = 0.5^{|j - k |}$. We let $\boldsymbol{w}_{i} = \boldsymbol{x}_{i}$ so that there is no exclusion restriction. For each $\boldsymbol{w}_{i}$, we generated values of $y_{i}$ and $s_{i}$, and repeated this for 1000 Monte Carlo replicates (keeping the covariates fixed for each scenario). We consider sample sizes $n = 500$ and 1000, and dimensions $p = 10, 25$ and 50, We performed simulations for $\rho = 0, 0.3, 0.5$ and 0.7, and found that the performance of each method was not dependent on $\rho$. For this reason, we only present the results for $\rho = 0.5$ in the main text, and include results for other values of $\rho$ in Appendix G. We compare the following variable selection methods:

1. A normal-normal Class I spike-and-slab prior, with hyperparameters elicited as in Table 1, using Beta-Binomial hyperparameters (1, 1).
2. A Laplace-Laplace Class I spike-and-slab prior, with hyperparameters as in the normal-normal case, choosing the scale parameter of the Laplace distributions such that they have equal variances to the normal case.
3. Adaptive LASSO as in Ogundimu (2022).
4. A vanilla implementation of forward selection which starts from the null model and, at each step, fits the maximum likelihood estimates of the parameters and adds the variable which minimizes the Bayesian Information Criterion (BIC) to either the selection or outcome equation (whichever minimizes the BIC).

### Performance measures

The evaluation metrics we use are: Sensitivity (TPR) - the proportion of active variables correctly identified; Specificity (TNR) - the proportion of inactive variables correctly identified; True model rate (TMR) - the proportion of replicates where the selected model is the true model; Model size - the average size of the selected model. For spike-and-slab samplers, the “selected model” is the median model, that is the model such that a variable is included if and only if the proportion of sampled models a variable is included in, also known as the posterior inclusion probability (PIP) is greater than 0.5. Formally, a variable $\beta _{j}$ is “included” if more than half of the posterior samples of $\gamma ^{O}_{j}$ are equal to 1, and similarly for $\alpha _{k}$. Barbieri and Berger (2004) shows in the normal linear case that this is the optimal predictive model.

We run the Gibbs samplers for 10,000 iterations, discarding the first 1, 250 iterations as burn-in. These choices provide a compromise between convergence of the chain and computational efficiency for 1,000 replicates in each simulation scenario. This is enough to stabilize the median model used to evaluate performance, but due to slow mixing of the chain (as further discussed in Appendix I), in real applications we recommend running the chain for longer, e.g. 50,000 iterations as in Sect. 6. The sampleSelection R package is used to choose initial values for the spike-and-slab samplers. That is, the parameter estimates from the maximum likelihood fit are used as the starting parameters, and the initial value of $\boldsymbol{\gamma }^{S}$ and $\boldsymbol{\gamma }^{O}$ are chosen such that a variable is included in the model if and only if its parameter estimate (under the maximum likelihood fit) is significant at a $5\%$ level. For both the normal and Laplace cases, we choose $\tau =5$ for the prior on ${\tilde{\rho }}$, *Beta*(1, 1) for the prior on *r*, and use the same distribution for the intercept as the rest of the model, with the chosen slab variance as its variance. For the normal case, we let $\tau _{1,\alpha } = \tau _{1,\beta } = 0.5$, and we divide these by $\sqrt{2}$ for the Laplace case.

### Convergence issues

In some instances, the maximum likelihood estimates can fail to converge for sample selection models. This can occur due to the non-convexity in the likelihood surface, potentially leading to convergence to local maxima (Olsen 1982), or issues with exclusion restrictions. While the model may be theoretically identifiable, in practice there is often practical non-identifiability for small sample sizes, large $\rho$ and large numbers of variables, especially in the presence of collinearity (Puhani 2000).

This is a particularly significant problem for the implementation of Adaptive LASSO as in Ogundimu (2022), which relies on a least-squares approximation of the likelihood of the *full* model around the maximum likelihood estimator. If maximum likelihood estimation fails then the algorithm breaks down. In each simulation scenario, we produced simulation replicates for Adaptive LASSO until we had 1, 000 where the MLE of the full model converged. For $n=1,000$ few extra replicates were required, but for $n=500$ there were issues. For $\rho = 0$, 37.2% of cases failed to converge; for $\rho = 0.3$, 51.8% of cases failed; for $\rho = 0.5$, 71.3% cases failed; and for $\rho = 0.7$, 91.8% of cases failed.

In contrast, the spike-and-slab sampler can still be run in these scenarios. As such, we used the original 1, 000 replicates, including replicates where the MLE failed to converge, for the spike-and-slab simulations, and this should be taken into account when comparing the results. Our initialization in cases where the MLE failed to converge was 0 for all model parameters aside from $\sigma$ which we set to 1.

The stepwise estimator also exhibited occasional issues with convergence, but less often than Adaptive LASSO, due to starting from a null model and on average including less variables than the Adaptive LASSO.

### Results

The results of the simulations can be found in Appendix F. Table 8 shows the performance of each method for $n=500$ and $p = 10, 25$ and 50 respectively. It should be noted that the medium and large effects are included with probability close to one, so that the sensitivity is almost entirely determined by the inclusion of the small effect. For $p=10$, the performance of the normal spike-and-slab sampler and the stepwise selection method are close. The normal spike-and-slab elicitation attains higher inclusion rates of small effects at the cost of lower specificity, while stepwise selection does the opposite. The Laplace spike-and-slab elicitation includes slightly more variables than the normal elicitation, while having lower specificity. The Adaptive LASSO performs similarly to stepwise in the outcome equation but struggles with false positives in the selection part of the equation, with an average model size of 3.458. We emphasize that these results for spike-and-slab priors depend on prior elicitation. For example, a larger slab variance for either prior distribution would improve specificity but exclude the small effect more often.

As the sparsity of the true model increases, the performance of every method deteriorates. The methods based on spike-and-slab priors exclude the small effect more often with increasing sparsity; this is a result of the multiple testing penalty imposed by the Beta-Binomial prior. Both Adaptive LASSO and stepwise selection struggle particularly as the sparsity increases. For $p=50$, the model size of Adaptive LASSO in the selection equation averages 4.57 when the true model size is 3, and it increasingly excludes the small effect in the outcome equation as *p* increases. Stepwise selection performs better but still has issues with false positives as *p* increases, averaging model sizes in each equation greater than 3.4 when $p=50$. Both spike-and-slab elicitations attain substantially larger true model rates and sensitivity than each of the Adaptive LASSO and stepwise methods for $p=25$ and $p=50$.

Table 9 compares the methods for $n=1000$. All methods perform significantly better, with Adaptive LASSO producing a competitive performance at this sample size. Adaptive LASSO exhibits better performance for the outcome equation in low dimensions, while the spike-and-slab priors outperform Adaptive LASSO in the presence of sparsity when taking the selection equation into account. Stepwise selection still attains strong performance for $p=10$ but as with $n=500$, the performance is significantly worse as *p* increases.

## Real data applications

In this section, we present two real data applications that illustrate the use of the proposed Bayesian variable selection methodology and how it compares against adaptive LASSO and stepwise selection. The first example analyzes ambulatory expenditures (Cameron and Trivedi 2010), whereas the second example analyzes data from the RAND Health Insurance Experiment (RAND HIE), a comprehensive study on the effect of health insurance on medical expenditures. For these applications, we standardize all the covariates and examine the parameter estimates and standard deviations using the standardized data, as the true models are unknown. For the spike-and-slab methods, we additionally look at the posterior inclusion probabilities and use the same priors as in Table 1, using Beta-Binomial elicitation $(1,p+q)$ (to account for low sparsity). The samplers are run for 50, 000 iterations, with the first 5, 000 being burn-in. We report the posterior median of the parameters, based on the proposed spike-and-slab priors, as a Bayes’ estimate. We additionally report the results from fitting the full model using the sampleSelection R package. We present the results for Class II priors and additional results in the Appendix. We briefly consider post-selection inference. To compare models without re-fitting the data, we follow the approach of Liang et al. (2008) by comparing parameter posteriors conditional on the given models, i.e. $\left( \boldsymbol{\alpha }, \boldsymbol{\beta }, \sigma , \rho \right) \mid \boldsymbol{\gamma }$. In our case we consider the sub-sample where the sampled model is $\boldsymbol{\gamma }$ (and only considering parameters with $\gamma _{j} = 1$). We evaluate the performance of different models using the Bayesian leave-one-out estimate of the expected log pointwise predictive density, referred to as $elpd_{loo}$ (Vehtari et al. 2017). We use the loo package in R to compute this.

### Ambulatory expenditures data

The ambulatory expenditures data contains information about several explanatory variables, such as age, gender, education status (educ), ethnicity (blhisp), number of chronic conditions (totchr), insurance status (ins) and income. The main aim is to study the effect of these variables on ambulatory expenditures. Not all patients had money spent, so that there are missing outcomes, and since we expect the decision to spend to be linked with the cost, this is a case of sample selection bias. The dataset contains $n=3,328$ observations, of which 516 ($15.8\%$) have missing expenditure. This dataset has been studied in previous publications (Marchenko and Genton 2012; Ogundimu 2022), and in line with these references we use log-expenditure (lambexp) as the outcome variable. We choose the same predictors as in previous publications, for comparison. That is, we let $\boldsymbol{x} = (\texttt {age}, \texttt {female}, \texttt {educ}, \texttt {blhisp}, \texttt {totchr}, \texttt {ins})^{\top }$ and $\boldsymbol{w} = (\boldsymbol{x}^{\top }, \texttt {income})^{\top }$ so that income is an exclusion restriction. The use of the income for this purpose is questionable (Cameron and Trivedi 2010), and an advantage of using variable selection methods in this context is to determine whether such restrictions are necessary.

Table 2 shows the results of applying each method to the data. The variables selected by the spike-and-slab model and stepwise are identical, and Adaptive LASSO selects similarly. All three methods exclude educ and ins from the outcome equation, with posterior inclusion probabilities being small for both variables. The major difference is in the exclusion restriction. The Adaptive LASSO still includes the exclusion restriction income, albeit shrunk more than the other included variables. The spike-and-slab normal, on the other hand, excludes income from the selection equation, with posterior inclusion probability 0.349. It is still included in a considerable number of posterior samples, so that the posterior distribution is bimodal, but the median estimate of the parameter is very close to zero as it lies in the spike part of the posterior instead of the slab. The variable ins is also somewhat shrunk in the selection equation, with inclusion probability 0.571. The larger standard deviations in each case is due to the bimodality of the posterior when the inclusion probability is close to 0.5.

**Table 2:** Results from ambulatory data

	Spike-and-slab normal			ALASSO		Stepwise		Full model	
	PIP	Est	S.D	Est	S.D	Est	S.D	Est	S.D
	Selection equation								
(Intercept)	-	1.276	0.038	1.270	0.038	1.278	0.038	1.283	0.038
educ	1.000	0.177	0.032	0.158	0.030	0.186	0.029	0.159	0.031
age	0.949	0.112	0.039	0.089	0.030	0.111	0.030	0.099	0.031
income	0.349	0.007	0.042	0.054	0.034	0.000	0.000	0.072	0.035
female	1.000	0.320	0.031	0.320	0.030	0.321	0.030	0.331	0.030
totchr	1.000	0.602	0.055	0.609	0.055	0.612	0.055	0.615	0.055
blhisp	1.000	−0.171	0.029	−0.161	0.028	−0.170	0.028	−0.168	0.029
ins	0.571	0.048	0.046	0.062	0.030	0.085	0.030	0.082	0.030
	Outcome equation								
(Intercept)	-	6.563	0.060	6.585	0.054	6.545	0.055	6.514	0.055
educ	0.116	0.004	0.018	0.000	0.000	0.000	0.000	0.048	0.027
age	1.000	0.230	0.026	0.222	0.026	0.232	0.026	0.238	0.026
female	1.000	0.158	0.032	0.141	0.030	0.165	0.030	0.174	0.030
totchr	1.000	0.399	0.031	0.390	0.030	0.406	0.030	0.417	0.030
blhisp	0.895	−0.094	0.039	−0.076	0.028	−0.103	0.028	−0.101	0.028
ins	0.033	−0.001	0.008	0.000	0.000	0.000	0.000	−0.014	0.025
$\sigma$	-	1.286	0.024	1.281	0.024	1.277	0.021	1.271	0.018
$\rho$	-	−0.265	0.154	−0.318	0.139	−0.215	0.145	−0.131	0.147

ALASSO refers to Adaptive LASSO, Stepwise refers to forward selection and “Full model” to the full model fit using the sampleSelection package. “PIP” refers to posterior inclusion probabilities for each variable, “Est.” refers to parameter estimates (posterior medians for spike-and-slab) and “S.D.” refers to standard deviations of the parameter estimates

Another difference is in the estimation of $\rho$. The full model estimates a significantly smaller value of $\rho$ compared to the Adaptive LASSO estimates and posterior median of the spike-and-slab normal. The spike-and-slab normal priors and Adaptive LASSO produce estimates of $\rho$ further away from zero.

For post-selection inference, we compared the spike-and-slab median model and Adaptive LASSO model using samples from the spike-and-slab sampler, and also include the model returning the lowest $elpd_{loo}$ of the 10 most sampled models, with results reported in table 3. The best model and Adaptive LASSO include more variables than spike-and-slab, but the gain in predictive performance is relatively small, suggesting these additional variables contribute only modestly to predictive power.

**Table 3:** Values of $elpd_{loo}$ of various models for the ambulatory data

	$elpd_{loo}$	Model size
Best model	−5086.0	12
Adaptive LASSO model	−5087.5	11
Spike-and-slab median model	−5087.7	10

The $elpd_{loo}$ for each model was computed using only parameter samples corresponding to iterations where the given model was sampled. The “best model” refers to the model with the lowest $elpd_{loo}$ of the 10 most sampled models by the spike-and-slab sampler

### RAND data

We now analyze a data set from the RAND Health Insurance Experiment (RAND HIE). This is a comprehensive study from 1971 to 1986 of the impact of randomized health insurance on health care cost, utilization and outcomes. This data set was used by Cameron and Trivedi (2005) to analyze how the patient’s use of health services is affected by types of randomly assigned health insurance. In line with previous applications, we use the variable lnmeddol, the logarithm of medical expenses per individual, as the outcome variable. We let $\boldsymbol{x}$ consist of the logarithm of coinsurance rate plus 1 ($\texttt {logc} = \log (\texttt {coins}+1)$), the dummy variable for individual deductible plan (idp), the logarithm of participation incentive payment (lpi), an artificial variable fmde that is 0 if $\texttt {idp} = 1$ and $\log (\max (1,\texttt {mde}/(0.01*\texttt {coins}))$ otherwise (where mde is the maximum expenditure offer), physical limitations (physlm), the number of chronic diseases (disea), dummy variables for good (hlthg), fair (hlthf) and poor (hlthp) self-rated health (where the baseline is excellent self-rated health), the log of family income (linc), the log of family size (lfam), education of household head in years (educdec), age of individual in years (xage), a dummy variable for female individuals (female), a dummy variable for individuals younger than 18 years (child), a dummy variable for female individuals younger than 18 years (fchild), and a dummy variable for black household heads (black). The selection variable is binexp which indicates whether the medical expenses are positive, and let $\boldsymbol{w} = \boldsymbol{x}$ so that there is no exclusion restriction. A subsample is selected so that the study year is 2 and educdec is not “NA”. There are 5, 574 observations, of which 1, 293 have zero medical expenses so that lnmeddol is missing. The table 7 contains the results of each method applied to the subsample. All the methods estimate similar values for $\rho$ of around 0.73, aside from stepwise selection which estimates $\rho = -0.225$. Stepwise selection also selects a completely different model, for instance including idp in the selection equation which is excluded from both spike-and-slab and Adaptive LASSO models, but excluding logc from the outcome equation when it has inclusion probability 1.000 in the spike-and-slab model and a large coefficient in the Adaptive LASSO model. The model selected by stepwise selection attains higher BIC than the model selected by the spike-and-slab sampler when the maximum likelihood estimates are fit to both models, suggesting that the search method has not been able to identify the optimal model for this dataset. The inference is otherwise similar between the normal spike-and-slab prior and Adaptive LASSO. In the selection equation, the same variables are excluded. The only difference in this regard is that the normal spike-and-slab prior excludes $\texttt {hlthg}$ from the outcome, with the inclusion probability being 0.217, whereas the Adaptive LASSO does not exclude this variable, though it does heavily shrink it to almost the same extent as the normal spike-and-slab prior. We also observe that the normal spike-and-slab prior produces larger estimates of the coefficients than Adaptive LASSO does.

**Fig. 2:**
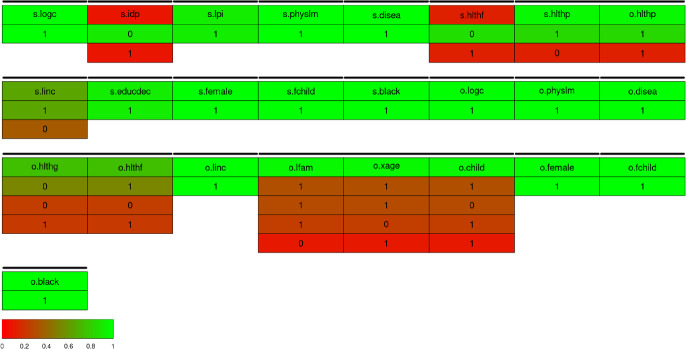
Visualisation of a 50% credible set for the model space posterior. The black bars above variables indicate groups - if a line is not broken between two variables, they are considered to be in the same group. The numbers indicate whether a variable is included or not and the color indicates the posterior inclusion probability of each listed group permutation

We take advantage of recent advances in Griffin (2024) to visualize the posterior in the model space. Figure 2 shows a 50% credible set for the model space posterior. Of most interest from this visualisation is the relation of the group consisting of lfam, xage and child, in particular the latter two. xage and child have a correlation of $-0.804$, and from Fig. 2, it is clear that there is significant overlap in the information each variable brings into the model, as all three combinations involving one (or both) have similar posterior inclusion probability. In this case, both variables have inclusion probabilities greater than 0.5, but if a higher cutoff were to be used, the visualisation suggests that at least one of these variables should be included regardless. It is also worth noting that hlthg and hlthf are grouped by this visualisation, which is where the only difference between the Adaptive LASSO model and the median model of the spike-and-slab occurs.

For post-selection inference, we compare the spike-and-slab median model and Adaptive LASSO model, using samples from the spike-and-slab sampler to obtain $elpd_{loo}$ values as in table 4. The best model of the 20 most sampled models in this case coincides with the model chosen by Adaptive LASSO, which includes one additional variable, but the gain in predictive performance by including this variable is modest.

**Table 4:** Values of $elpd_{loo}$ of various models for the RAND data

	$elpd_{loo}$	Model size
Adaptive LASSO model (best model)	−8541.6	23
Spike-and-slab median model	−8548.2	22

The $elpd_{loo}$ for each model was computed using only parameter samples corresponding to iterations where the given model was sampled. The “best model” refers to the model with the lowest $elpd_{loo}$ of the 20 most sampled models by the spike-and-slab sampler

### Sensitivity analysis

In this section, we perform a sensitivity analysis for the spike and slab variances on the two datasets.

Let $\tau _{1,\alpha }^{*} = \sqrt{3}/\pi$ and $\tau _{1,\beta }^{*} = (4\log (500))^{-1/2}(\log (n))^{1/2}$. Alongside those values, which are the defaults for the previous analysis, we consider two additional specifications. We consider $\tau _{1,\alpha } = 2\tau _{1,\alpha }^{*}$ and $\tau _{1,\beta } = 2\tau _{1,\beta }^{*}$, alongside the choices $\tau _{1,\alpha } = 0.5\tau _{1,\alpha }^{*}$ and $\tau _{1,\beta } = 0.5\tau _{1,\beta }^{*}$. In each case, the variances have been multiplied or shrunk by a factor of 4, so that the values have change significantly. Other than the choice of spike-and-slab variances, we use the same elicitation as in the previous two sections. Table 20 shows the comparison between the three different choices on the ambulatory data. Similar tables for the spike variance and analogous tables for the RAND data can also be found in the table in Appendix J. As expected, the larger slab variances have smaller inclusion probabilities for every variable. Despite the difference in prior variances, though, the difference between the models is not drastic. The only difference in the maximum a posteriori model is the exclusion of ins in the selection equation by the larger slab variances. The posterior median of the coefficients for variables with inclusion probability not close to 1 are shrunk more by the larger variances, but otherwise are similar. Similar behaviour occurs for the RAND data (table 22). Due to the high collinearity between the predictors, some variables actually have higher inclusion probability under the larger slab variance, when highly correlated variables are excluded in their place. Tables 21 and 23 show that posterior inference based on the spike-and-slab prior is less sensitive to the spike variance than the slab variance. There is little difference in inclusion probability for most variables.

## Conclusion

We proposed two continuous spike-and-slab prior structures for variable selection in the context of sample selection models, and provided practical guidelines for calibrating these priors. We developed Gibbs samplers, with closed-form tractable conditionals, for sample selection models coupled with the proposed prior structures. An appealing feature of the proposed prior structures and Gibbs samplers is that they allow for using (different) scale mixture of normals for the spike and slab components. We have shown that these Gibbs samplers are scalable to the dimensions of interest in practice. The time complexity is not linear, as clearly seen in Appendix A, so it will not scale well to dimensions $p> > 200$. However, applications of interest studied in sample selection literature are seldom higher dimensional than this.

Our simulation study shows that the proposed Bayesian variable selection methodology offers a good performance in terms of sensitivity and specificity of the selected variables. Moreover, it exhibits similar performance to Adaptive LASSO in low dimensions and large sample sizes, while identifying the true model more frequently for extremely sparse models and smaller sample sizes. A significant advantage of our proposed methodology is that, unlike Adaptive LASSO, our proposed methodology does not suffer from convergence issues for moderately small sample sizes and higher dimensions.

There exist several natural extensions of our work. It is desirable to extend theoretical results, similar to Narisetty and He (2014), to sample selection models, and to use these results to inform prior elicitation, or use a data-driven approach to elicitate the hyperparameters instead (Tadesse and Vannucci 2021). Regarding scalability, one could extend the Skinny Gibbs algorithm (Narisetty et al. 2019) to sample selection, which would let the algorithm scale to higher dimensions, or to develop *ad hoc* variational Bayes methods (Tadesse and Vannucci 2021). A similar approach could be taken with EM algorithms, such as the spike-and-slab LASSO by Ročková and George (2018). We assumed bivariate normal errors in the two-equation model (1). It would be possible to extend this model to non-normal errors, such as scale mixtures of normals (or other distributions with a tractable stochastic representation), which would allow to use the Gibbs samplers developed in Sect. 4 with additional steps. The proposed Gibbs samplers can also be extended to the case with binary outcomes, by using the stochastic representation of probit models through latent variables. The focus in this paper is primarily on model selection and the problem of post-selection inference requires further investigation. Because the continuous spike-and-slab returns posterior probabilities for each model, an extension of the methodology in this paper could incorporate Bayesian model averaging (Raftery et al. 1997; Steel 2020) into the output of the sampler directly. The issue of excluded parameters having positive posterior density away from zero under continuous spike-and-slab priors would need to be carefully considered in this context.

## Figures and Tables

**Fig. 3:**
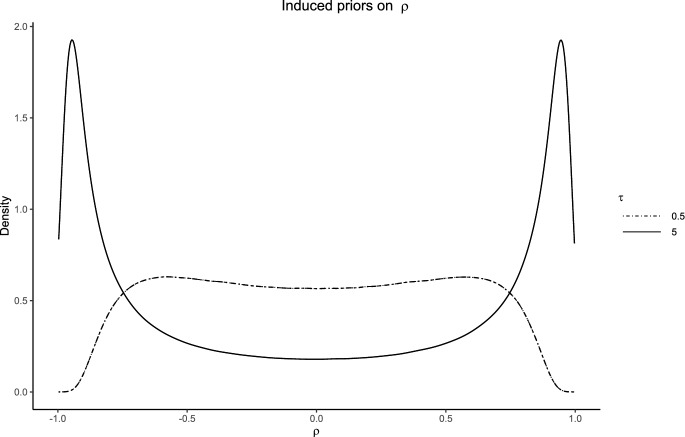
The induced priors on $\rho$ for $\tau = 5$ and 0.5 respectively. Smaller choices of $\tau$ are closer to a uniform distribution, while larger choices resemble Beta distributions, except approaching zero at the tails

**Fig. 4:**
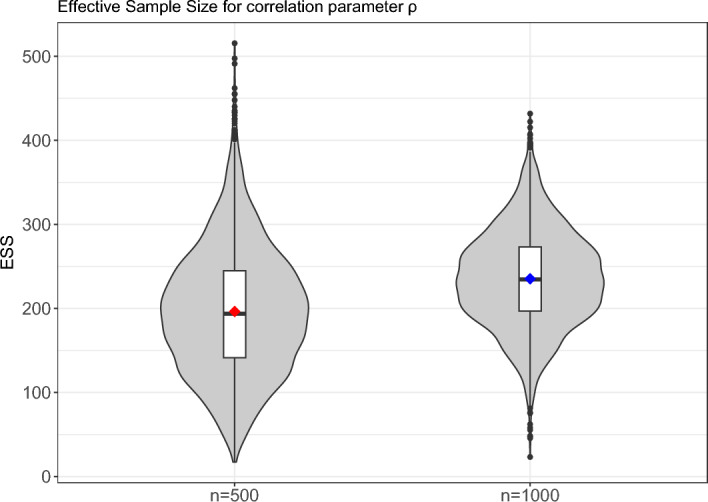
Violin plot of effective sample sizes for $\rho$ across simulation replicates

**Fig. 5:**
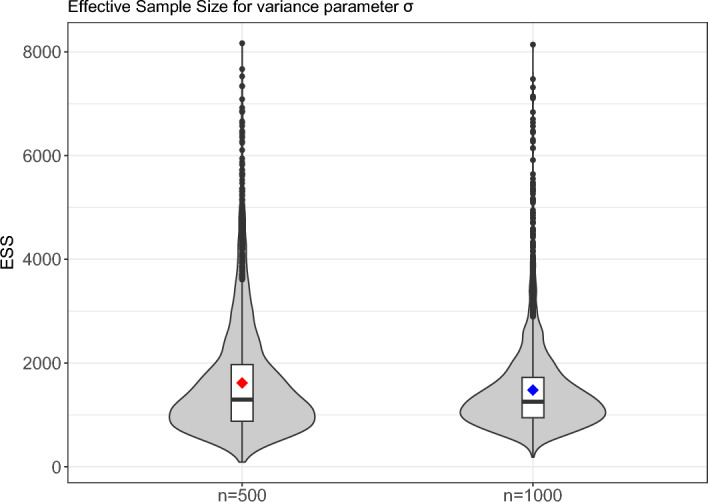
Violin plot for effective sample sizes of $\sigma$ across simulation replicates

**Fig. 6:**
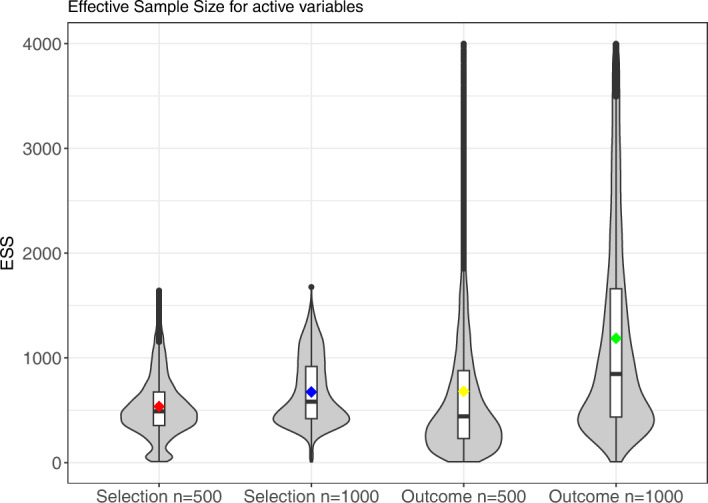
Violin plot for effective sample sizes of active variables across simulation replicates, with each equation pooled separately. The long tail for $n=1000$ outside the graph has been cut off for clarity

**Fig. 7:**
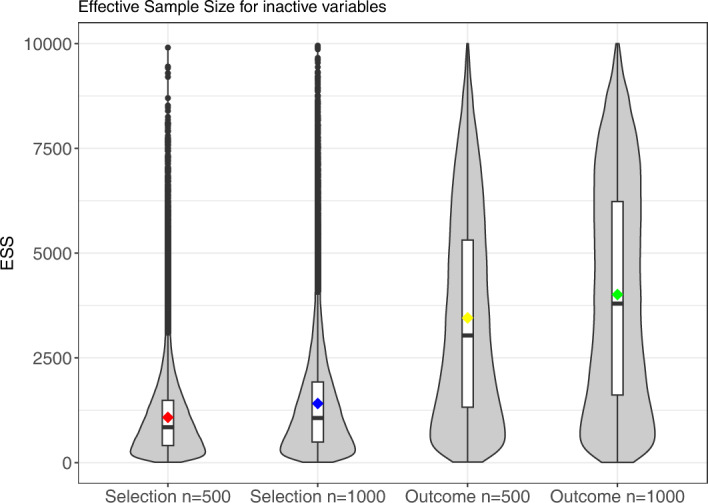
Violin plot for effective sample sizes of inactive variables across simulation replicates, with each equation pooled separately. The long tail for $n=1000$ outside the graph has been cut off for clarity

## Appendix A Computational times

To test computational times for the spike-and-slab Gibbs sampler, we recorded the running time of the method on one replicate from the simulation study. That is, we simulated $\boldsymbol{x}_{i} = (x_{i,1}, \ldots , x_{i,p})^{\top }$ as i.i.d. normal for $i = 1, \ldots , n$, let $\boldsymbol{w} = \boldsymbol{x}$, then generated bivariate normal errors to obtain $(y_{i}, s_{i})^{\top }$ from (1), with $\sigma =1$. We chose $\rho = 0.5$ and to avoid convergence issues, $n=1000$. For the spike-and-slab normal, the chain was run for 10, 000 iterations, discarding the first 1, 250 as burn-in, as in the simulation study. We varied *p* from 25 to 200 (and let $q = p$) to see how the running times scale to very high dimensions. The computations were performed on a computer with 16GB of RAM and an AMD Ryzen 5 3600 GPU. The reason this was run separately from the rest of the simulation study is because the original simulations were run in parallel and only considered up to $p=50$ Table 5.

**Table 5:** Computational times for the spike-and-slab prior, for $n=1000$

Method	$p=25$	$p=50$	$p=100$	$p=200$
Spike-and-slab normal	15.5	39.3	130.7	609.0

All times are in seconds

## Appendix B Induced prior for $\rho$

Recall that ${\tilde{\sigma }}^{2} \sim IG(c,d)$ and $({\tilde{\rho }} \mid {\tilde{\sigma }}^{2}) \sim N(0, \tau {\tilde{\sigma }}^{2})$, with $c, d, \tau > 0$ positive constants. $\tau$ controls the behaviour of the induced prior on $\rho$. Figure 3 shows the shape of two induced priors on (0, 1), one with $\tau = 0.5$ and the other with $\tau = 5$. The case $\tau =5$ closely resembles a *Beta*(1/2, 1/2) prior, while $\tau =0.5$ resembles a uniform prior, aside from both approaching zero at the bounds 0 and 1.

Alongside the simulation study in Sect. 5, we ran the same simulations for the normal-normal spike-and-slab sampler with $\tau = 0.5$ (and otherwise the same choices of prior parameters). Table 6 shows how the posterior median of $\rho$ differed between these two priors. Both priors underestimate $\rho$, but the prior with $\tau = 5$ consistently gives estimates closer to the true value. The posterior inference for other parameters (not shown here) is similar for both choices of $\tau$, so we prefer $\tau = 5$ to 0.5.

**Table 6:** Comparison of posterior median of $\rho$ for $\tau =0.5$ and $\tau =5$

n	True $\rho$	$\tau =0.5$	$\tau =5$
500	0	−0.112	−0.128
500	0.3	0.132	0.148
500	0.5	0.323	0.363
500	0.7	0.558	0.615
1000	0	−0.028	−0.030
1000	0.3	0.246	0.259
1000	0.5	0.442	0.464
1000	0.7	0.647	0.674

Uses spike-and-slab normal priors for each case, with the same elicitation and same 1000 replicates as in the simulation study. The posterior medians of $\rho$ are very similar for all *p*, so we only include $p=10$ for brevity

## Appendix C Gibbs sampler for sample selection models

Here, we reproduce the Gibbs sampler from (van Hasselt 2011) for completeness. Let the priors on the parameters be

$$\begin{aligned} (\alpha _{0},\boldsymbol{\alpha }^{\top })^{\top }&\sim N(\boldsymbol{a},\boldsymbol{A}) \, ,\\ (\beta _{0},\boldsymbol{\beta }^{\top })^{\top }&\sim N(\boldsymbol{b}, \boldsymbol{B}) \, ,\\ {\tilde{\rho }} \mid {\tilde{\sigma }}&\sim N(0, \tau {\tilde{\sigma }}^{2}) \, ,\\ {\tilde{\sigma }}^{2}&\sim \text {IG}(c,d) \, . \end{aligned}$$

where $\boldsymbol{a} \in {\mathbb {R}}^{p+1}$, $\boldsymbol{A} \in {\mathbb {R}}^{(p+1)\times (p+1)}$, $\boldsymbol{b} \in {\mathbb {R}}^{q+1}$, $\boldsymbol{B} \in {\mathbb {R}}^{(q+1)\times (q+1)}$, $\tau \in (0,1)$ and $c, d > 0$ are real constants.

Further define $\boldsymbol{X} = (\boldsymbol{1},\boldsymbol{x}_{1},\ldots ,\boldsymbol{x}_{n})$ and $\boldsymbol{W} = (\boldsymbol{1},\boldsymbol{w}_{1},\ldots ,\boldsymbol{w}_{n})$. Let $\boldsymbol{W}_{0}$ be the design matrix $\boldsymbol{W}$ with only the observations that have missing $y_{i}$, and $\boldsymbol{W}_{1}$ the design matrix with observations that have non-missing $y_{i}$. Similarly, let $\boldsymbol{X}_{1}$ be the design matrix $\boldsymbol{X}$ with the observations that have non-missing $y_{i}$, $\boldsymbol{y}_{1}$ be the non-missing outcomes, $\boldsymbol{s^{*}}_{1}$ the corresponding values of $s^{*}$ and $\boldsymbol{s^{*}}_{0}$ the values of $s^{*}$ for observations with missing outcomes. Furthermore, let $\boldsymbol{g} = (\boldsymbol{\beta }^{\top }, 0)^{\top }$ and

$$\begin{aligned} \boldsymbol{G} = \begin{pmatrix} \boldsymbol{B} & \boldsymbol{0}\\ \boldsymbol{0} & \tau {\tilde{\sigma }}^{2} \end{pmatrix} , \end{aligned}$$

and let $\boldsymbol{Z}$ be the design matrix $\boldsymbol{X}_{1}$ with an additional column $\boldsymbol{s^{*}}_{1} - \boldsymbol{W}_{1}\boldsymbol{\alpha }$. Then the Gibbs sampler as in van Hasselt (2011) is as follows:

1. Sample from $\boldsymbol{s^{*}}$, where for $i = 1, \ldots , n$ where $s^{*}_{i} \mid \{y_{i}, s_{i}, \alpha _{0}, \beta _{0}, \boldsymbol{\alpha }, \boldsymbol{\beta }, {\tilde{\rho }}, {\tilde{\sigma }}\}$ is distributed according to
  $$\begin{aligned} {\left\{ \begin{array}{ll} \mathcal{T}\mathcal{N}_{(-\infty , 0)}(\alpha _{0} + \boldsymbol{w}_{i}^{\top }\boldsymbol{\alpha }, 1) & \text {if } y_{i} \text { is missing},\\ \mathcal{T}\mathcal{N}_{(0,\infty )}\left( \alpha _{0} + \boldsymbol{w}_{i}^{\top }\boldsymbol{\alpha } + \frac{{\tilde{\rho }}}{{\tilde{\rho }}^{2} + {\tilde{\sigma }}^{2}}(y_{i} - \beta _{0} - \boldsymbol{x}_{i}^{\top }\boldsymbol{\beta }), \frac{{\tilde{\sigma }}^{2}}{{\tilde{\rho }}^{2} + {\tilde{\sigma }}^{2}}\right) & \text {otherwise} \end{array}\right. } \end{aligned}$$
  where $\mathcal{T}\mathcal{N}_{(a,b)}$ denotes a truncated normal distribution on (*a*, *b*).
2. Sample $(\alpha _{0},\boldsymbol{\alpha }^{\top })^{\top }$ from $N(\boldsymbol{a^{*}}, \boldsymbol{A^{*}})$, where
  $$\begin{aligned} \boldsymbol{A^{*}}&= \left( \boldsymbol{A}^{-1} + \boldsymbol{W}_{0}^{\top }\boldsymbol{W}_{0} + \left( \frac{{\tilde{\sigma }}^{2}}{{\tilde{\sigma }}^{2} + {\tilde{\rho }}^{2}}\right) ^{-1}\boldsymbol{W}_{1}^{\top }\boldsymbol{W}_{1}\right) ^{-1} \, ,\\ \boldsymbol{a^{*}}&= \boldsymbol{A^{*}}\left( \boldsymbol{A}^{-1}\boldsymbol{a} + \boldsymbol{W}_{0}^{\top }\boldsymbol{s^{*}}_{0} + \left( \frac{{\tilde{\sigma }}^{2}}{{\tilde{\sigma }}^{2} + {\tilde{\rho }}^{2}}\right) ^{-1}\boldsymbol{W}_{1}^{\top }\left( \boldsymbol{s^{*}}_{1} - \frac{{\tilde{\rho }}}{{\tilde{\sigma }}^{2} + {\tilde{\rho }}^{2}}(\boldsymbol{y}_{1} - \boldsymbol{X}_{1}\boldsymbol{\beta })\right) \right) \, . \end{aligned}$$
3. Jointly sample $(\beta _{0},\boldsymbol{\beta }^{\top }, {\tilde{\rho }})^{\top }$ from $N(\boldsymbol{b^{*}}, \boldsymbol{B^{*}})$, where
  $$\begin{aligned} \boldsymbol{B^{*}}&= \left( \boldsymbol{G}^{-1} + \frac{1}{{\tilde{\sigma }}^{2}}\boldsymbol{Z}^{\top }\boldsymbol{Z}\right) ^{-1} \, ,\\ \boldsymbol{b^{*}}&= \boldsymbol{B^{*}}\left( \boldsymbol{G}^{-1}\boldsymbol{g} + \frac{1}{{\tilde{\sigma }}^{2}}\boldsymbol{Z}^{\top }\boldsymbol{y}_{1}\right) \, . \end{aligned}$$
4. Sample ${\tilde{\sigma }}^{2}$ from $\text {IG}(c^{*},d^{*})$, where
  $$\begin{aligned} c^{*}&= c + \frac{1}{2}\left( 1 + \sum _{i=1}^{n} s_{i}\right) \, ,\\ d^{*}&= d + \frac{{\tilde{\rho }}^{2}}{2\tau } + \frac{1}{2}\sum _{\{i: s_{i}=1\}}\left( y_{i} - \beta _{0} - \boldsymbol{x}_{i}{^\top }\boldsymbol{\beta } - {\tilde{\rho }}(s^{*}_{i} - \alpha _{0} -\boldsymbol{w}_{i}^{\top }\boldsymbol{\alpha })\right) ^{2} \, . \end{aligned}$$

## Appendix D Further details on the Gibbs samplers

### D.1 Class I prior

The prior variance matrix for the outcome equation, $\boldsymbol{B}$, corresponds to the $(p+1) \times (p+1)$ diagonal matrix where $B_{1,1} = \eta ^{O} v^{O}_{0}$ and $B_{j,j} = \left( (1 - \gamma ^{O}_{j-1})\tau _{0,\beta }^{2}v + \gamma ^{O}_{j-1}\tau _{1,\beta }^{2}\right) v^{O}_{j-1}$ (with the mixing variable $v^{O}_{j-1}$ appearing to allow for scale mixture of normal priors). $\boldsymbol{A}$ is defined similarly. Define $\boldsymbol{W}_{0}, \boldsymbol{W}_{1}, \boldsymbol{s^{*}}_{0}, \boldsymbol{s^{*}}_{1}, \boldsymbol{y}_{1}, \boldsymbol{g}, \boldsymbol{G}$ and $\boldsymbol{Z}$ as in Section C. Then we have that

$$\begin{aligned} \boldsymbol{A^{*}}&= \left( \boldsymbol{A}^{-1} + \boldsymbol{W}_{0}^{\top }\boldsymbol{W}_{0} + \left( \frac{{\tilde{\sigma }}^{2}}{{\tilde{\sigma }}^{2} + {\tilde{\rho }}^{2}}\right) ^{-1}\boldsymbol{W}_{1}^{\top }\boldsymbol{W}_{1}\right) ^{-1} \, ,\\ \boldsymbol{a^{*}}&= \boldsymbol{A^{*}}\left( \boldsymbol{W}_{0}^{\top }\boldsymbol{s^{*}}_{0} + \left( \frac{{\tilde{\sigma }}^{2}}{{\tilde{\sigma }}^{2} + {\tilde{\rho }}^{2}}\right) ^{-1}\boldsymbol{W}_{1}^{\top }\left( \boldsymbol{s^{*}}_{1} - \frac{{\tilde{\rho }}}{{\tilde{\sigma }}^{2} + {\tilde{\rho }}^{2}}(\boldsymbol{y} - \boldsymbol{X}\boldsymbol{\beta })\right) \right) \, ,\\ \boldsymbol{B^{*}}&= \left( \boldsymbol{G}^{-1} + \frac{1}{{\tilde{\sigma }}^{2}}\boldsymbol{Z}^{\top }\boldsymbol{Z}\right) ^{-1} \, ,\\ \boldsymbol{b^{*}}&= \boldsymbol{B^{*}}\left( \frac{1}{{\tilde{\sigma }}^{2}}\boldsymbol{Z}^{\top }\boldsymbol{y}_{1}\right) \, . \end{aligned}$$

### D.2 Class II prior

The Gibbs sampler for the Class II prior is similar to the Class I prior, with only two differences. The first difference is that wherever $\tau _{0,\beta }$ and $\tau _{1,\beta }$ appear, $\tau _{0,\beta }{\tilde{\sigma }}$ and $\tau _{1,\beta }{\tilde{\sigma }}$ appear instead. The second difference are the values of $c^{*}$ and $d^{*}$ in sampling ${\tilde{\sigma }}^{2}$ (in Step 4 of the algorithm). That is, we instead have that

$$\begin{aligned} c^{*}&= c + \frac{1}{2}\left( 1 + \sum _{i=1}^{n} s_{i}\right) + \frac{p+1}{2}\, ,\\ d^{*}&= d + \frac{{\tilde{\rho }}^{2}}{2\tau } + \frac{1}{2}\sum _{\{i: s_{i}=1\}}\left( y_{i} - \beta _{0} - \boldsymbol{x}_{i}{^\top }\boldsymbol{\beta } - {\tilde{\rho }}(s^{*}_{i} - \alpha _{0} - \boldsymbol{w}_{i}^{\top }\boldsymbol{\alpha })^{2}\right) + \frac{\beta _{0}^{2}}{2\eta ^{O} v^{O}_{0}} \\&+ \sum _{j=1}^{p} \frac{\beta _{j}^{2}}{2v^{O}_{j}((1-\gamma ^{O}_{j})\tau _{0,\beta }^{2} + \gamma ^{O}_{j}\tau _{1,\beta }^{2})}\, . \end{aligned}$$

## Appendix E RAND data table

**Table 7:** Results from RAND health data. ALASSO refers to Adaptive LASSO, Stepwise refers to forward selection and “Full model” to the full model fit using the sampleSelection package

	Spike-and-slab normal			ALASSO		Stepwise		Full model	
	PIP	Est	S.D	Est	S.D	Est	S.D	Est	S.D
	Selection equation								
(Intercept)	-	0.813	0.020	0.801	0.020	0.819	0.020	0.815	0.020
logc	1.000	−0.229	0.030	−0.198	0.022	−0.218	0.023	−0.218	0.054
idp	0.094	−0.001	0.012	0.000	0.000	−0.062	0.020	−0.048	0.022
lpi	0.919	0.072	0.028	0.043	0.020	0.079	0.022	0.079	0.023
fmde	0.090	0.001	0.023	0.000	0.000	0.000	0.000	0.003	0.055
physlm	0.998	0.099	0.024	0.073	0.022	0.100	0.023	0.092	0.023
disea	1.000	0.154	0.023	0.150	0.023	0.169	0.024	0.143	0.024
hlthg	0.020	0.000	0.004	0.000	0.000	0.000	0.000	0.028	0.021
hlthf	0.131	0.001	0.018	0.000	0.000	0.000	0.000	0.060	0.022
hlthp	0.844	0.077	0.038	0.053	0.023	0.000	0.000	0.099	0.025
linc	0.640	0.048	0.034	0.024	0.020	0.052	0.020	0.068	0.020
lfam	0.019	0.000	0.005	0.000	0.000	0.000	0.000	−0.017	0.022
educdec	0.922	0.071	0.027	0.050	0.019	0.087	0.021	0.089	0.021
xage	0.031	0.000	0.007	0.000	0.000	0.000	0.000	−0.010	0.035
female	1.000	0.196	0.024	0.164	0.023	0.209	0.024	0.205	0.027
child	0.057	0.001	0.011	0.000	0.000	0.000	0.000	0.026	0.039
fchild	1.000	−0.143	0.024	−0.111	0.022	−0.154	0.023	−0.157	0.031
black	1.000	−0.227	0.021	−0.223	0.019	−0.230	0.019	−0.225	0.020
	Outcome equation								
(Intercept)	-	3.550	0.039	3.561	0.038	4.124	0.096	3.543	0.036
logc	1.000	−0.229	0.027	−0.209	0.024	0.000	0.000	−0.155	0.069
idp	0.033	−0.001	0.007	0.000	0.000	0.000	0.000	−0.066	0.029
lpi	0.046	0.001	0.010	0.000	0.000	0.000	0.000	0.040	0.028
fmde	0.037	0.000	0.011	0.000	0.000	−0.116	0.022	−0.081	0.067
physlm	1.000	0.124	0.025	0.102	0.024	0.080	0.023	0.115	0.024
disea	1.000	0.205	0.027	0.204	0.026	0.127	0.024	0.194	0.026
hlthg	0.236	0.002	0.026	0.026	0.023	0.000	0.000	0.075	0.025
hlthf	0.740	0.066	0.040	0.060	0.023	0.000	0.000	0.120	0.026
hlthp	0.964	0.100	0.031	0.086	0.023	0.085	0.021	0.124	0.023
linc	0.997	0.138	0.034	0.108	0.027	0.109	0.026	0.148	0.028
lfam	0.853	−0.079	0.037	−0.060	0.024	−0.077	0.024	−0.086	0.027
educdec	0.076	0.001	0.014	0.000	0.000	0.000	0.000	0.050	0.026
xage	0.749	0.115	0.071	0.100	0.037	0.104	0.037	0.096	0.041
female	1.000	0.279	0.035	0.252	0.031	0.148	0.030	0.275	0.032
child	0.670	−0.110	0.083	−0.109	0.043	−0.136	0.044	−0.097	0.048
fchild	1.000	−0.234	0.044	−0.204	0.037	−0.122	0.036	−0.224	0.039
black	1.000	−0.205	0.030	−0.197	0.029	0.000	0.000	−0.207	0.029
$\sigma$	-	1.571	0.029	1.561	0.029	1.410	0.018	1.570	0.028
$\rho$	-	0.729	0.039	0.721	0.038	−0.225	0.076	0.736	0.034

## Appendix F Main simulation tables

### F.1 $n=500$

**Table 8:** Evaluation metrics for $n=500, \rho =0.5$. “TMR” refers to the proportion of seeds where the true model was selected and “Size” to the number of variables in the model

Method	Selection equation				Outcome equation			
	TMR	Size	Sens	Spec	TMR	Size	Sens	Spec
	$$p=10$$							
Normal	0.783	3.173	0.985	0.969	0.794	3.045	0.970	0.981
Laplace	0.669	3.402	0.991	0.939	0.730	3.219	0.982	0.961
$\hbox {Stepwise}^{*}$	0.831	2.990	0.968	0.988	0.808	2.957	0.960	0.989
$\hbox {ALASSO}^{*}$	0.558	3.458	0.982	0.927	0.762	2.919	0.945	0.988
	$$p=25$$							
Normal	0.767	3.043	0.964	0.993	0.777	2.938	0.950	0.996
Laplace	0.728	3.139	0.971	0.990	0.755	3.026	0.959	0.993
$\hbox {Stepwise}^{*}$	0.682	3.198	0.970	0.987	0.699	3.176	0.969	0.988
$\hbox {ALASSO}^{*}$	0.420	3.900	0.980	0.956	0.702	2.874	0.927	0.996
	$$p=50$$							
Normal	0.723	2.942	0.940	0.997	0.657	2.805	0.906	0.998
Laplace	0.717	3.007	0.948	0.997	0.685	2.888	0.924	0.998
$\hbox {Stepwise}^{*}$	0.511	3.498	0.968	0.987	0.534	3.429	0.964	0.989
$\hbox {ALASSO}^{*}$	0.271	4.570	0.973	0.965	0.537	2.717	0.871	0.998

Stepwise uses forward selection and ALASSO refers to Adaptive LASSO. $^{*}$ Performance measures for Adaptive LASSO and stepwise selection were only evaluated over replicates that converged and had finite variance

$^{*}$ Performance measures for Adaptive LASSO and stepwise selection were only evaluated over replicates that converged and had finite variance

### F.2 $n=1000$

**Table 9:** Evaluation metrics for $n=1000, \rho =0.5$

Method	Selection equation				Outcome equation			
	TMR	Size	Sens	Spec	TMR	Size	Sens	Spec
	$$p=10$$							
Normal	0.885	3.129	1.000	0.981	0.932	3.074	1.000	0.989
Laplace	0.815	3.224	1.000	0.968	0.874	3.142	1.000	0.980
$\hbox {Stepwise}^{*}$	0.925	3.064	0.998	0.990	0.942	3.059	1.000	0.991
$\hbox {ALASSO}^{*}$	0.877	3.123	0.999	0.982	0.974	2.980	0.992	1.000
	$$p=25$$							
Normal	0.911	3.091	0.999	0.996	0.935	3.056	0.998	0.997
Laplace	0.891	3.121	0.999	0.994	0.898	3.100	0.999	0.995
$\hbox {Stepwise}^{*}$	0.837	3.182	1.000	0.992	0.833	3.177	1.000	0.992
$\hbox {ALASSO}^{*}$	0.650	3.493	1.000	0.978	0.938	3.033	0.995	0.998
	$$p=50$$							
Normal	0.904	3.086	0.998	0.998	0.945	3.042	0.997	0.999
Laplace	0.880	3.125	0.999	0.997	0.922	3.070	0.997	0.998
$\hbox {Stepwise}^{*}$	0.667	3.405	1.000	0.991	0.698	3.384	1.000	0.992
$\hbox {ALASSO}^{*}$	0.476	3.833	0.999	0.982	0.938	3.009	0.991	0.999

“TMR” refers to the proportion of seeds where the true model was selected and “Size” to the number of variables in the model. Stepwise uses forward selection and ALASSO refers to Adaptive LASSO

$^{*}$ Performance measures for Adaptive LASSO and stepwise selection were only evaluated over replicates that converged and had finite variance

## Appendix G Additional tables for different correlations

### G.1 $n=500$

See Tables 10, 11, 12, 13, 14, 15, 16, 17

**Table 10:** Evaluation metrics for $n=500, \rho =0$

Method	Selection equation				Outcome equation			
	TMR	Size	Sens	Spec	TMR	Size	Sens	Spec
	$$p=10$$							
Normal	0.804	3.156	0.987	0.972	0.783	3.031	0.965	0.980
Laplace	0.660	3.412	0.992	0.938	0.719	3.222	0.980	0.960
$\hbox {Stepwise}^{*}$	0.855	3.007	0.975	0.988	0.808	2.938	0.955	0.990
$\hbox {ALASSO}^{*}$	0.561	3.535	0.991	0.920	0.753	2.941	0.947	0.986
	$$p=25$$							
Normal	0.783	3.046	0.967	0.993	0.760	2.927	0.945	0.996
Laplace	0.757	3.136	0.973	0.990	0.745	3.04	0.961	0.993
$\hbox {Stepwise}^{*}$	0.691	3.192	0.970	0.987	0.690	3.154	0.966	0.988
$\hbox {ALASSO}^{*}$	0.388	4.010	0.986	0.952	0.691	2.890	0.928	0.995
	$$p=50$$							
Normal	0.725	3.020	0.952	0.997	0.680	2.843	0.917	0.998
Laplace	0.699	3.092	0.958	0.995	0.700	2.927	0.933	0.997
$\hbox {Stepwise}^{*}$	0.495	3.562	0.972	0.986	0.520	3.450	0.965	0.988
$\hbox {ALASSO}^{*}$	0.242	4.704	0.982	0.963	0.643	2.865	0.912	0.997

“TMR” refers to the proportion of seeds where the true model was selected and “Size” to the number of variables in the model. Stepwise uses forward selection and ALASSO refers to Adaptive LASSO

$^{*}$ Performance measures for Adaptive LASSO and stepwise selection were only evaluated over replicates that converged and had finite variance

**Table 11:** Evaluation metrics for $n=500, \rho =0.3$

Method	Selection equation				Outcome equation			
	TMR	Size	Sens	Spec	TMR	Size	Sens	Spec
	$$p=10$$							
Normal	0.771	3.203	0.987	0.966	0.792	3.029	0.967	0.982
Laplace	0.658	3.439	0.992	0.934	0.700	3.237	0.978	0.957
$\hbox {Stepwise}^{*}$	0.820	3.003	0.968	0.986	0.809	2.962	0.960	0.988
$\hbox {ALASSO}^{*}$	0.554	3.509	0.984	0.920	0.764	2.941	0.948	0.986
	$$p=25$$							
Normal	0.754	3.053	0.964	0.993	0.766	2.946	0.948	0.995
Laplace	0.724	3.153	0.972	0.989	0.749	3.034	0.957	0.993
$\hbox {Stepwise}^{*}$	0.683	3.218	0.973	0.986	0.694	3.182	0.969	0.987
$\hbox {ALASSO}^{*}$	0.404	3.980	0.983	0.953	0.690	2.880	0.924	0.995
	$$p=50$$							
Normal	0.716	2.965	0.941	0.997	0.677	2.804	0.910	0.998
Laplace	0.706	3.038	0.951	0.996	0.691	2.897	0.926	0.997
$\hbox {Stepwise}^{*}$	0.506	3.525	0.970	0.987	0.532	3.433	0.965	0.989
$\hbox {ALASSO}^{*}$	0.264	4.609	0.976	0.964	0.567	2.750	0.881	0.998

“TMR” refers to the proportion of seeds where the true model was selected and “Size” to the number of variables in the model. Stepwise uses forward selection and ALASSO refers to Adaptive LASSO

$^{*}$ Performance measures for Adaptive LASSO and stepwise selection were only evaluated over replicates that converged and had finite variance

**Table 12:** Evaluation metrics for $n=500, \rho =0.7$

Method	Selection equation				Outcome equation			
	TMR	Size	Sens	Spec	TMR	Size	Sens	Spec
	$$p=10$$							
Normal	0.786	3.169	0.985	0.969	0.828	3.036	0.975	0.984
Laplace	0.699	3.369	0.991	0.943	0.761	3.190	0.984	0.966
$\hbox {Stepwise}^{*}$	0.813	2.969	0.961	0.988	0.804	2.945	0.956	0.989
$\hbox {ALASSO}^{*}$	0.612	3.343	0.977	0.941	0.779	2.905	0.946	0.991
	$$p=25$$							
Normal	0.750	3.027	0.959	0.993	0.782	2.939	0.952	0.996
Laplace	0.730	3.115	0.967	0.990	0.766	3.019	0.962	0.994
$\hbox {Stepwise}^{*}$	0.668	3.199	0.965	0.986	0.698	3.166	0.967	0.988
$\hbox {ALASSO}^{*}$	0.426	3.788	0.973	0.961	0.713	2.895	0.932	0.996
	$$p=50$$							
Normal	0.726	2.938	0.94	0.997	0.705	2.816	0.919	0.999
Laplace	0.711	3.002	0.947	0.997	0.703	2.898	0.930	0.998
$\hbox {Stepwise}^{*}$	0.500	3.503	0.966	0.987	0.512	3.410	0.959	0.989
$\hbox {ALASSO}^{*}$	0.223	4.689	0.965	0.962	0.515	2.667	0.859	0.998

“TMR” refers to the proportion of seeds where the true model was selected and “Size” to the number of variables in the model. Stepwise uses forward selection and ALASSO refers to Adaptive LASSO

$^{*}$ Performance measures for Adaptive LASSO and stepwise selection were only evaluated over replicates that converged and had finite variance

### G.2 $n=1000$

**Table 13:** Evaluation metrics for $n=1000, \rho =0$

Method	Selection equation				Outcome equation			
	TMR	Size	Sens	Spec	TMR	Size	Sens	Spec
	$$p=10$$							
Normal	0.895	3.112	0.999	0.984	0.927	3.079	1.000	0.989
Laplace	0.833	3.189	1.000	0.973	0.870	3.149	1.000	0.979
$\hbox {Stepwise}^{*}$	0.932	3.049	0.997	0.992	0.948	3.054	1.000	0.992
$\hbox {ALASSO}^{*}$	0.876	3.122	0.999	0.982	0.959	2.971	0.988	0.999
	$$p=25$$							
Normal	0.908	3.093	0.998	0.996	0.936	3.043	0.996	0.997
Laplace	0.857	3.160	0.999	0.993	0.909	3.084	0.998	0.996
$\hbox {Stepwise}^{*}$	0.825	3.192	0.999	0.991	0.830	3.177	0.998	0.992
$\hbox {ALASSO}^{*}$	0.582	3.580	0.999	0.974	0.927	3.026	0.992	0.998
	$$p=50$$							
Normal	0.919	3.071	0.998	0.998	0.933	3.049	0.996	0.999
Laplace	0.891	3.105	0.998	0.998	0.896	3.096	0.997	0.998
$\hbox {Stepwise}^{*}$	0.694	3.354	1.000	0.992	0.680	3.382	0.999	0.992
$\hbox {ALASSO}^{*}$	0.443	3.937	1.000	0.980	0.930	3.028	0.992	0.999

“TMR” refers to the proportion of seeds where the true model was selected and “Size” to the number of variables in the model. Stepwise uses forward selection and ALASSO refers to Adaptive LASSO

$^{*}$ Performance measures for Adaptive LASSO and stepwise selection were only evaluated over replicates that converged and had finite variance

**Table 14:** Evaluation metrics for $n=1000, \rho =0.3$

Method	Selection equation				Outcome equation			
	TMR	Size	Sens	Spec	TMR	Size	Sens	Spec
	$$p=10$$							
Normal	0.880	3.121	0.999	0.982	0.928	3.074	0.999	0.989
Laplace	0.812	3.209	0.999	0.970	0.878	3.135	1.000	0.981
$\hbox {Stepwise}^{*}$	0.931	3.056	0.998	0.991	0.942	3.055	0.999	0.992
$\hbox {ALASSO}^{*}$	0.853	3.146	0.998	0.978	0.973	2.983	0.993	0.999
	$$p=25$$							
Normal	0.917	3.082	0.999	0.996	0.919	3.066	0.997	0.997
Laplace	0.886	3.125	1.000	0.994	0.892	3.107	0.999	0.995
$\hbox {Stepwise}^{*}$	0.828	3.183	1.000	0.992	0.825	3.178	0.999	
$\hbox {ALASSO}^{*}$	0.619	3.519	1.000	0.976	0.929	3.035	0.993	0.998
	$$p=50$$							
Normal	0.903	3.088	0.998	0.998	0.939	3.036	0.995	0.999
Laplace	0.873	3.123	0.998	0.997	0.923	3.060	0.996	0.998
$\hbox {Stepwise}^{*}$	0.664	3.398	0.999	0.991	0.679	3.383	0.999	0.992
$\hbox {ALASSO}^{*}$	0.458	3.906	1.000	0.981	0.940	3.017	0.992	0.999

“TMR” refers to the proportion of seeds where the true model was selected and “Size” to the number of variables in the model. Stepwise uses forward selection and ALASSO refers to Adaptive LASSO. $^{*}$ Performance measures for Adaptive LASSO and stepwise selection were only evaluated over replicates that converged and had finite variance

$^{*}$ Performance measures for Adaptive LASSO and stepwise selection were only evaluated over replicates that converged and had finite variance

**Table 15:** Evaluation metrics for $n=1000, \rho =0.7$

Method	Selection equation				Outcome equation			
	TMR	Size	Sens	Spec	TMR	Size	Sens	Spec
	$$p=10$$							
Normal	0.899	3.116	1.000	0.983	0.938	3.064	1.000	0.991
Laplace	0.839	3.201	1.000	0.971	0.878	3.133	1.000	0.981
$\hbox {Stepwise}^{*}$	0.939	3.059	0.999	0.991	0.940	3.057	0.999	0.992
$\hbox {ALASSO}^{*}$	0.915	3.080	0.998	0.988	0.984	2.986	0.995	1.000
	$$p=25$$							
Normal	0.921	3.079	0.999	0.996	0.943	3.048	0.998	0.998
Laplace	0.877	3.132	1.000	0.994	0.914	3.084	0.999	0.996
$\hbox {Stepwise}^{*}$	0.830	3.182	1.000	0.992	0.827	3.188	1.000	0.991
$\hbox {ALASSO}^{*}$	0.667	3.449	0.999	0.980	0.945	3.035	0.997	0.998
	$$p=50$$							
Normal	0.911	3.089	0.999	0.998	0.940	3.048	0.997	0.999
Laplace	0.882	3.126	1.000	0.997	0.924	3.075	0.998	0.998
$\hbox {Stepwise}^{*}$	0.668	3.399	1.000	0.992	0.688	3.387	1.000	0.992
$\hbox {ALASSO}^{*}$	0.477	3.847	0.999	0.982	0.934	3.029	0.993	0.999

“TMR” refers to the proportion of seeds where the true model was selected and “Size” to the number of variables in the model. Stepwise uses forward selection and ALASSO refers to Adaptive LASSO. $^{*}$ Performance measures for Adaptive LASSO and stepwise selection were only evaluated over replicates that converged and had finite variance

$^{*}$ Performance measures for Adaptive LASSO and stepwise selection were only evaluated over replicates that converged and had finite variance

## Appendix H Class II prior comparison

Recall that ${\tilde{\sigma }}^{2} = \sigma ^{2}(1-\rho ^{2})$. For $\rho = 0$, the Class II prior is exactly dependent on the outcome variance, and the prior is shrunk for $|\rho |> 0$. In the simulation studies, we chose $\sigma ^{2} = 1$, so the Class II prior performs similarly to the Class I prior for small $\rho$; for this reason, we present results for the most extreme case, $\rho = 0.7$.

**Table 16:** Comparison between Class I and Class II priors for $n=500, \rho =0.7$

Method	Selection equation				Outcome equation			
	TMR	Size	Sens	Spec	TMR	Size	Sens	Spec
	$$p=10$$							
Class I normal	0.786	3.169	0.985	0.969	0.828	3.036	0.975	0.984
Class II normal	0.787	3.191	0.987	0.967	0.790	3.122	0.980	0.974
Class I Laplace	0.699	3.369	0.991	0.943	0.761	3.190	0.984	0.966
Class II Laplace	0.674	3.45	0.993	0.933	0.676	3.362	0.987	0.943
	$$p=25$$							
Class I normal	0.750	3.027	0.959	0.993	0.782	2.939	0.952	0.996
Class II normal	0.760	3.044	0.963	0.993	0.774	2.984	0.957	0.995
Class I Laplace	0.730	3.115	0.967	0.990	0.766	3.019	0.962	0.994
Class II Laplace	0.732	3.128	0.969	0.990	0.753	3.073	0.967	0.992
	$$p=50$$							
Class I normal	0.726	2.938	0.940	0.997	0.705	2.816	0.919	0.999
Class II normal	0.745	2.952	0.947	0.998	0.705	2.858	0.924	0.998
Class I Laplace	0.711	3.002	0.947	0.997	0.703	2.898	0.930	0.998
Class II Laplace	0.717	3.035	0.953	0.996	0.719	2.915	0.935	0.998

“TMR” refers to the proportion of seeds where the true model was selected and “Size” to the number of variables in the model

**Table 17:** Comparison between Class I and Class II priors for $n=1000, \rho =0.7$

Method	Selection equation				Outcome equation			
	TMR	Size	Sens	Spec	TMR	Size	Sens	Spec
	$$p=10$$							
Class I normal	0.899	3.116	1.000	0.983	0.938	3.064	1.000	0.991
Class II normal	0.893	3.125	1.000	0.982	0.902	3.104	1.000	0.985
Class I Laplace	0.839	3.201	1.000	0.971	0.878	3.133	1.000	0.981
Class II Laplace	0.813	3.235	1.000	0.966	0.813	3.224	1.000	0.968
	$$p=25$$							
Class I normal	0.921	3.079	0.999	0.996	0.943	3.048	0.998	0.998
Class II normal	0.915	3.086	0.999	0.996	0.922	3.068	0.998	0.997
Class I Laplace	0.877	3.132	1.000	0.994	0.914	3.084	0.999	0.996
Class II Laplace	0.867	3.15	1.000	0.993	0.885	3.116	0.999	0.995
	$$p=50$$							
Class I normal	0.911	3.089	0.999	0.998	0.940	3.048	0.997	0.999
Class II normal	0.921	3.081	1.000	0.998	0.920	3.078	0.998	0.998
Class I Laplace	0.882	3.126	1.000	0.997	0.924	3.075	0.998	0.998
Class II Laplace	0.872	3.141	1.000	0.997	0.903	3.102	0.999	0.998

“TMR” refers to the proportion of seeds where the true model was selected and “Size” to the number of variables in the model

The two perform fairly similarly, with Class II priors including more variables on average than Class I priors (reflected in the model size).

## Appendix I MCMC diagnostics

To assess convergence of the Gibbs sampler, we look at the Effective Sample Size (ESS) of parameter estimates for different replicates in the Simulation study. For each of the 1, 000 replicates in the main simulation study scenarios (Section F, i.e. $\rho = 0.5$), we recorded the ESS of each variable, with the total chain length being 8, 750. For each variable, we pooled results for $p=10, 25$ and 50 as we found that the ESS did not differ significantly with number of variables. For simplicity of presentation, we pool the active variables in each equation together, and inactive variables in each equation together, alongside presenting the ESS of $\rho$.

Figure 4 shows the ESS of $\rho$ for $n=500$ and $n=1000$. The median ESS is just below 200 for $n=500$, which is small relative to the chain length of 8, 750. This suggests that the mixing is very slow. It should be noted that $\rho$ is a particularly difficult variable to estimate in sample selection models: it typically has a large standard error (such as in the ambulatory data: see Table 2) while also having issues with convergence of maximum likelihood estimation (see 5.3). For instance, in one case where $\rho$ has an ESS of just 23, the cause is severe bimodality in the true model from the generated data. Regardless, the Gibbs sampler still mixes slowly, even for $n=1,000$ (where the median ESS is still below 250).

It should also be noted that $\rho$ and $\sigma$ are not sampled directly, but instead ${\tilde{\rho }} =\rho \sigma$ and ${\tilde{\sigma }} = \sqrt{\sigma ^{2}(1-\rho ^{2})}$. Figure 5 shows that the effective sample sizes for $\sigma$ are significantly larger than those for $\rho$, but internally (not shown here) we found that the effective sample sizes for ${\tilde{\rho }}$ and ${\tilde{\sigma }}$ were closer to that of $\rho$ than $\sigma$. This suggests that the problematic part of the error distribution to model is not the variance but the correlation. Additionally, the ratio of effective sample size to chain length is consistent with those reported by Wiemann et al. (2022), suggesting that the problem may be inherent to $\rho$.

Figures 6 and 7 show the ESS values for active and inactive variables respectively in each equation. In each case, the graph is cut off for clarity (there is a long but narrow tail in each $n=1000$ case). Unsurprisingly, the mixing in the selection equation is on average slower than the mixing in the outcome equation, while the median ESS varies from around 500 to almost 4, 000, with inactive variables having higher ESS than active ones.

Aside from the active variables in the selection equation, there is a not-insignificant density towards very low ESS values. There are two causes of this. Firstly, as $\rho$ changes values, so will the distribution of the other parameters conditional on $\rho$. Because $\rho$ mixes slowly, it will slow down the mixing for other active parameters too. This is particularly true for the intercept and large effects in the outcome equation: $\rho \sigma$ is sampled as a “covariate” alongside the outcome parameters in the Gibbs sampler in Section C, so any large effects have highly correlated parameter samples. As such, a small effective sample size for $\rho$ will lead to small effective sample sizes for other correlated parameters. Secondly, for variables that have posterior inclusion probability not close to 0 or 1, the posterior will be bimodal (though the mode corresponding to the slab can be very small if the posterior inclusion probability is small, e.g. around 0.2). This is exacerbated by the correlation of the parameter with $\rho$ when it is included in the model, leading to very slow mixing around one of the modes.

It should also be noted that there are cases where the effective sample size exceeds the chain length (8, 750) for spurious variables. Gibbs samplers cannot be theoretically antithetic, but sometimes finite sample autocorrelation can be negative. For some spurious variables with very low inclusion probability, this can occur by chance.

For the simulation study, the performance measures were taken on the median model. In particular, the median model only differs if a posterior inclusion probability changes from one side of 0.5 to the other. We found that in practice, that posterior inclusion probabilities did not drastically differ even when effective sample sizes were small and mixing was slow (potentially because the areas of highest posterior density are those being sampled frequently) so that 10, 000 iterations was sufficient to stabilize the median model. We prioritized additional Monte Carlo replicates to reduce variation caused by different data samples, as opposed to variance caused by chains. To verify this, we performed the simulation study in Section F for $n=500$, $p=10$ only, with a chain length of 50, 000 instead of 10, 000, and a thinning interval of 5 for memory purposes. We found that the results differed very marginally between the chain lengths.

Regardless, for practical applications we would suggest running the chain for longer, e.g. $n=50,000$ as in Sect. 6. With the computational times reported in Appendix A for 10, 000 iterations, this is not infeasible and should allow for an effective sample size of all parameters above 500. Furthermore, diagnosing convergence of the chain can be done post-hoc: more iterations can easily be added if necessary Table 18.

**Table 18:** Comparison of different chain lengths for $n=500$, $p=10$, $\rho = 0.5$ for spike-and-slab priors

Method	Selection equation				Outcome equation			
	TMR	Size	Sens	Spec	TMR	Size	Sens	Spec
Normal 10, 000	0.784	3.162	0.984	0.970	0.797	3.045	0.971	0.981
Normal 50, 000	0.783	3.173	0.985	0.969	0.794	3.045	0.970	0.981
Laplace 10, 000	0.669	3.402	0.991	0.939	0.730	3.219	0.982	0.960
Laplace 50, 000	0.666	3.402	0.991	0.939	0.732	3.215	0.982	0.962

For chain length 50, 000, a thinning interval of 5 was used

Finally, we test the impact of different initial values by running a small simulation study for $n=500, p=10, \rho =0.5$, we simulate the data as before, but run 10 chains (each for 10, 000 and discarding 1, 250 as burn-in) from different initial values. We record the point-estimate of the Gelman-Rubin statistic ${\widehat{R}}$ taken for these 10 chains. We repeat this for 100 replicates and summarize the quantiles of the sampled ${\widehat{R}}$ below.

To choose random initial values, we chose an initial model at complete random and then sampled initial values for active coefficients from normal distributions (appropriately transforming $\sigma$ and $\rho$ to be in $(0,\infty )$ and $(-1,1)$ respectively). It should be noted that this is an extreme scenario as the initial model may not only include many irrelevant variables but also may exclude variables with very large effect sizes and coefficients may have completely opposite signs to their true values, as opposed to initializing around the MLE which is more likely to start in a region of high posterior density.

Table 19 shows that the ${\widehat{R}}$ is very close to 1 for almost all simulation replicates, which suggests the 10 chains converged to the same distribution for almost all replicates. The only variable that displays a departure from this is in the variable $\rho$, where a single replicate has its ${\widehat{R}}$ exceed 1.05. Given the potentially extreme initial values used and the lower value of ${\widehat{R}}$ for $\rho$ in all other replicates, we do not think this is problematic, but this can be remedied by running the chain for, say, 50, 000 replicates, which we suggest for all practical applications regardless.

**Table 19:** Table testing the impact of different initial values on point estimators of ${\widehat{R}}$

Variables	${\widehat{R}}$ point-estimate quantile						
	0%	2.5%	25%	50%	75%	97.5%	100%
Active $\boldsymbol{\alpha }$	1.000	1.001	1.002	1.002	1.004	1.009	1.012
Inactive $\boldsymbol{\alpha }$	1.000	1.001	1.002	1.003	1.004	1.008	1.020
Active $\boldsymbol{\beta }$	1.000	1.000	1.001	1.002	1.004	1.012	1.025
Inactive $\boldsymbol{\beta }$	1.000	1.000	1.000	1.001	1.001	1.003	1.005
$\rho$	1.002	1.002	1.004	1.006	1.010	1.024	1.053
$\sigma$	1.000	1.000	1.001	1.001	1.001	1.003	1.005

The point-estimates were recorded for 100 different replicates, hence the quantiles of the point-estimates being reported. $\boldsymbol{\alpha }$ refers to selection equation variables and $\boldsymbol{\beta }$ to outcome equation variables

## Appendix J Additional tables for sensitivity analysis

### J.1 Varying slab variance for ambulatory data

**Table 20:** Results from ambulatory data from varying the slab variances. The first column refers to what $\tau _{1,\alpha }$ and $\tau _{1,\beta }$ have been scaled by

Slab variance scaling	$\times 1$			$\times 0.25$			$\times 4$		
	PIP	Est	S.D	PIP	Est	S.D	PIP	Est	S.D
	Selection equation								
(Intercept)	-	1.276	0.038	-	1.268	0.037	-	1.275	0.038
educ	1.000	0.177	0.032	1.000	0.172	0.032	0.996	0.181	0.033
age	0.949	0.112	0.039	0.977	0.109	0.034	0.914	0.115	0.044
income	0.349	0.007	0.042	0.471	0.012	0.044	0.206	0.005	0.037
female	1.000	0.320	0.031	1.000	0.319	0.030	1.000	0.320	0.031
totchr	1.000	0.602	0.055	1.000	0.585	0.052	1.000	0.604	0.055
blhisp	1.000	−0.171	0.029	1.000	−0.169	0.029	1.000	−0.174	0.029
ins	0.571	0.048	0.046	0.693	0.062	0.044	0.388	0.009	0.044
	Outcome equation								
(Intercept)	-	6.563	0.060	-	6.562	0.057	-	6.566	0.059
educ	0.116	0.004	0.018	0.196	0.005	0.021	0.062	0.003	0.014
age	1.000	0.230	0.026	1.000	0.229	0.026	1.000	0.231	0.026
female	1.000	0.158	0.032	1.000	0.158	0.030	1.000	0.157	0.031
totchr	1.000	0.399	0.031	1.000	0.398	0.030	1.000	0.398	0.031
blhisp	0.895	−0.094	0.039	0.952	−0.095	0.033	0.844	−0.093	0.042
ins	0.033	−0.001	0.008	0.079	−0.002	0.010	0.017	−0.001	0.008
$\sigma$	-	1.286	0.024	-	1.286	0.023	-	1.287	0.024
$\rho$	-	−0.265	0.154	-	−0.266	0.145	-	−0.273	0.150

The elicitation is otherwise the same as the previous data studies

### J.2 Varying spike variance for ambulatory data

**Table 21:** Results from ambulatory data from varying the spike variances. The first column refers to what $\tau _{0,\alpha }$ and $\tau _{0,\beta }$ have been scaled by

Spike variances scaling	$\times 1$			$\times 0.25$			$\times 4$		
	PIP	Est	S.D	PIP	Est	S.D	PIP	Est	S.D
	Selection equation								
(Intercept)	-	1.276	0.038	-	1.278	0.038	-	1.275	0.039
educ	1.000	0.177	0.032	1.000	0.177	0.032	1.000	0.175	0.031
age	0.949	0.112	0.039	0.944	0.110	0.040	0.910	0.110	0.041
income	0.349	0.007	0.042	0.402	0.004	0.045	0.301	0.016	0.039
female	1.000	0.320	0.031	1.000	0.322	0.031	1.000	0.321	0.030
totchr	1.000	0.602	0.055	1.000	0.605	0.055	1.000	0.601	0.056
blhisp	1.000	−0.171	0.029	1.000	−0.171	0.029	1.000	−0.172	0.029
ins	0.571	0.048	0.046	0.626	0.056	0.046	0.487	0.031	0.042
	Outcome equation								
(Intercept)	-	6.563	0.060	-	6.556	0.059	-	6.564	0.060
educ	0.116	0.004	0.018	0.131	0.001	0.019	0.103	0.011	0.019
age	1.000	0.230	0.026	1.000	0.231	0.026	1.000	0.231	0.026
female	1.000	0.158	0.032	1.000	0.161	0.031	0.999	0.158	0.032
totchr	1.000	0.399	0.031	1.000	0.401	0.031	1.000	0.398	0.032
blhisp	0.895	−0.094	0.039	0.935	−0.097	0.036	0.816	−0.090	0.040
ins	0.033	−0.001	0.008	0.036	0.000	0.006	0.039	−0.004	0.013
$\sigma$	-	1.286	0.024	-	1.284	0.023	-	1.286	0.024
$\rho$	-	−0.265	0.154	-	−0.245	0.152	-	−0.268	0.155

The elicitation is otherwise the same as the previous data studies

### J.3 Varying slab for RAND data

**Table 22:** Results from RAND data from varying the slab variances. The first column refers to what $\tau _{1,\alpha }$ and $\tau _{1,\beta }$ have been scaled by

Slab variance scaling	$\times 1$			$\times 0.25$			$\times 4$		
	PIP	Est	S.D	PIP	Est	S.D	PIP	Est	S.D
	Selection equation								
(Intercept)	-	0.813	0.020	-	0.813	0.020	-	0.813	0.020
logc	1.000	−0.229	0.030	1.000	−0.226	0.033	1.000	−0.229	0.029
idp	0.094	−0.001	0.012	0.206	−0.002	0.018	0.047	−0.001	0.009
lpi	0.919	0.072	0.028	0.930	0.069	0.027	0.899	0.073	0.030
fmde	0.090	0.001	0.023	0.155	0.001	0.028	0.068	0.001	0.023
physlm	0.998	0.099	0.024	0.997	0.096	0.024	1.000	0.100	0.023
disea	1.000	0.154	0.023	1.000	0.152	0.023	1.000	0.155	0.023
hlthg	0.020	0.000	0.004	0.049	0.000	0.006	0.007	0.000	0.004
hlthf	0.131	0.001	0.018	0.336	0.003	0.026	0.051	0.001	0.011
hlthp	0.844	0.077	0.038	0.960	0.084	0.030	0.724	0.069	0.042
linc	0.640	0.048	0.034	0.849	0.059	0.029	0.552	0.037	0.035
lfam	0.019	0.000	0.005	0.046	0.000	0.006	0.009	0.000	0.004
educdec	0.922	0.071	0.027	0.961	0.073	0.024	0.833	0.067	0.032
xage	0.031	0.000	0.007	0.072	0.000	0.009	0.012	0.000	0.005
female	1.000	0.196	0.024	1.000	0.194	0.024	1.000	0.196	0.024
child	0.057	0.001	0.011	0.082	0.001	0.012	0.016	0.001	0.006
fchild	1.000	−0.143	0.024	1.000	−0.141	0.025	1.000	−0.143	0.023
black	1.000	−0.227	0.021	1.000	−0.223	0.020	1.000	−0.228	0.021
	Outcome equation								
(Intercept)	-	3.551	0.039	-	3.552	0.040	-	3.544	0.039
logc	1.000	−0.229	0.027	0.994	−0.226	0.036	1.000	−0.230	0.026
idp	0.033	−0.001	0.007	0.102	−0.001	0.015	0.013	0.000	0.005
lpi	0.046	0.001	0.010	0.085	0.001	0.012	0.022	0.000	0.007
fmde	0.037	0.000	0.011	0.106	0.000	0.024	0.020	0.000	0.009
physlm	1.000	0.124	0.025	1.000	0.121	0.025	1.000	0.129	0.025
disea	1.000	0.205	0.027	1.000	0.201	0.027	1.000	0.208	0.027
hlthg	0.236	0.002	0.026	0.497	0.008	0.033	0.084	0.001	0.017
hlthf	0.740	0.066	0.040	0.898	0.082	0.037	0.507	0.021	0.040
hlthp	0.964	0.100	0.031	0.993	0.108	0.026	0.886	0.092	0.038
linc	0.997	0.138	0.034	1.000	0.147	0.031	1.000	0.130	0.034
lfam	0.853	−0.079	0.037	0.916	−0.080	0.033	0.715	−0.072	0.043
educdec	0.076	0.001	0.014	0.175	0.001	0.021	0.036	0.001	0.010
xage	0.749	0.115	0.071	0.783	0.100	0.063	0.736	0.136	0.079
female	1.000	0.279	0.035	1.000	0.271	0.034	1.000	0.285	0.035
child	0.670	−0.110	0.083	0.799	−0.120	0.073	0.544	−0.079	0.092
fchild	1.000	−0.234	0.044	1.000	−0.222	0.042	1.000	−0.244	0.044
black	1.000	−0.205	0.030	1.000	−0.203	0.030	1.000	−0.207	0.030
$\sigma$	-	1.571	0.029	-	1.568	0.030	-	1.577	0.030
$\rho$	-	0.729	0.039	-	0.725	0.041	-	0.736	0.040

The elicitation is otherwise the same as the previous data studies

### J.4 Varying spike for RAND data

**Table 23:** Results from RAND data from varying the spike variances. The first column refers to what $\tau _{0,\alpha }$ and $\tau _{0,\beta }$ have been scaled by

Spike variance scaling	$\times 1$			$\times 0.25$			$\times 4$		
	PIP	Est	S.D	PIP	Est	S.D	PIP	Est	S.D
	Selection equation								
(Intercept)	-	0.813	0.020	-	0.813	0.020	-	0.813	0.020
logc	1.000	−0.229	0.030	1.000	−0.227	0.032	1.000	−0.229	0.032
idp	0.094	−0.001	0.012	0.115	−0.001	0.013	0.082	−0.004	0.012
lpi	0.919	0.072	0.028	0.897	0.069	0.029	0.870	0.071	0.030
fmde	0.090	0.001	0.023	0.118	0.000	0.027	0.111	0.002	0.027
physlm	0.998	0.099	0.024	0.981	0.098	0.027	0.988	0.099	0.025
disea	1.000	0.154	0.023	1.000	0.153	0.023	1.000	0.154	0.024
hlthg	0.020	0.000	0.004	0.016	0.000	0.003	0.019	0.000	0.007
hlthf	0.131	0.001	0.018	0.147	0.001	0.019	0.105	0.004	0.016
hlthp	0.844	0.077	0.038	0.916	0.081	0.033	0.809	0.075	0.038
linc	0.640	0.048	0.034	0.758	0.055	0.032	0.619	0.046	0.033
lfam	0.019	0.000	0.005	0.019	0.000	0.004	0.021	0.000	0.007
educdec	0.922	0.071	0.027	0.924	0.070	0.027	0.881	0.070	0.029
xage	0.031	0.000	0.007	0.033	0.000	0.006	0.028	−0.001	0.008
female	1.000	0.196	0.024	1.000	0.195	0.025	1.000	0.196	0.024
child	0.057	0.001	0.011	0.054	0.000	0.011	0.046	0.002	0.011
fchild	1.000	−0.143	0.024	1.000	−0.142	0.024	1.000	−0.144	0.024
black	1.000	−0.227	0.021	1.000	−0.225	0.021	1.000	−0.227	0.021
	Outcome equation								
(Intercept)	-	3.550	0.039	-	3.547	0.038	-	3.548	0.039
logc	1.000	−0.229	0.027	1.000	−0.230	0.027	0.998	−0.229	0.029
idp	0.033	−0.001	0.007	0.050	0.000	0.009	0.039	−0.002	0.010
lpi	0.046	0.001	0.010	0.047	0.000	0.009	0.044	0.002	0.011
fmde	0.037	0.000	0.011	0.045	0.000	0.012	0.044	0.000	0.015
physlm	1.000	0.124	0.025	0.998	0.123	0.026	0.999	0.124	0.026
disea	1.000	0.205	0.027	1.000	0.205	0.027	1.000	0.206	0.027
hlthg	0.236	0.002	0.026	0.304	0.001	0.029	0.220	0.006	0.026
hlthf	0.740	0.066	0.040	0.823	0.071	0.038	0.714	0.066	0.040
hlthp	0.964	0.100	0.031	1.000	0.104	0.025	0.968	0.100	0.030
linc	0.997	0.138	0.034	1.000	0.145	0.031	0.999	0.137	0.033
lfam	0.853	−0.079	0.037	0.924	−0.082	0.032	0.777	−0.075	0.040
educdec	0.076	0.001	0.014	0.088	0.000	0.015	0.070	0.003	0.014
xage	0.749	0.115	0.071	0.681	0.098	0.072	0.712	0.106	0.071
female	1.000	0.279	0.035	1.000	0.277	0.034	1.000	0.277	0.034
child	0.670	−0.110	0.083	0.735	−0.125	0.083	0.730	−0.123	0.081
fchild	1.000	−0.234	0.044	1.000	−0.229	0.044	1.000	−0.230	0.043
black	1.000	−0.205	0.030	1.000	−0.205	0.029	1.000	−0.206	0.030
$\sigma$	-	1.571	0.029	-	1.573	0.029	-	1.572	0.030
$\rho$	-	0.729	0.039	-	0.732	0.038	-	0.730	0.039

The elicitation is otherwise the same as the previous data studies
